# Strand-Exchange Nucleic Acid Circuitry with Enhanced Thermo-and Structure- Buffering Abilities Turns Gene Diagnostics Ultra-Reliable and Environmental Compatible

**DOI:** 10.1038/srep36605

**Published:** 2016-11-04

**Authors:** Zhentong Zhu, Yidan Tang, Yu Sherry Jiang, Sanchita Bhadra, Yan Du, Andrew D. Ellington, Bingling Li

**Affiliations:** 1 State Key Lab of Electroanalytical Chemistry, Changchun Institute of Applied Chemistry, Chinese Academy of Science, Changchun, Jilin, 130022, P.R. China; 2 University of Chinese Academy of Sciences, Beijing, 100039, China; 3 Center for Systems and Synthetic Biology, University of Texas at Austin, Austin, Texas 78712, United States

## Abstract

Catalytic hairpin assembly (CHA) is one of the most promising nucleic acid amplification circuits based on toehold-mediated strand exchange reactions. But its performance is usually ruined by fluctuated environmental temperatures or unexpected self-structures existing in most real-world targets. Here we present an amide-assistant mechanism that successfully reduces the prevalence of these problems for CHA and maximizes its thermo- and structure- buffering abilities. Such an organic amide-promoted CHA (shortened as OHT-CHA) can unprecedentedly amplify through 4 °C to 60 °C without rebuilding sequences or concerning target complexity. We are then for the first time able to employ it as a direct and universal signal booster for loop mediated isothermal reaction (LAMP). LAMP is one of the most promising point-of-care (POC) gene amplifiers, but has been hard to detect precisely due to structured products and haunted off-target amplicons. OHT-CHA guarantees a significant and reliable signal for LAMP reaction amplified from as little as 10^−19^ M virus gene. And one single set of OHT-CHA is qualified to any detection requirement, either in real-time at LAMP running temperature (~60 °C), or at end-point on a POC photon counter only holding environmental temperatures fluctuating between 4 °C to 42 °C.

Recent advances in the field of molecular computing have yielded a series of nucleic-acid-alone amplification circuits relying on parallel or series toehold-mediated strand exchange reactions^1^,^2^,^3^,^4^,^5^,^6^,^7^,^8^. One of these promising circuits, catalytic two hairpin assembly (known as CHA)^8^,^9^, uses a single stranded DNA oligonucleotide as an input/catalyst to initial two successive toehold-mediated stand exchange/displacement reactions that assemble two hairpin substrates into duplex products (Fig. 1A). Under a regular reaction condition, an ideal CHA reaction is capable of amplifying 50 to 100 fold within a few hours (Fig. 1B, column 1). Due to the high signal-to-background ratio and programmability, CHA has been widely applied into detecting various analytical platforms and considered as a new generation of signal amplifier and transducer. A wide range of targets have been designed as catalysts for detection, including oligonucleotides^10^,^11^, aptamer-intermediated non-nucleic acid species such as proteins and small molecules^12^,^13^, nucleic acid nanostructures^14^, and certain isothermal nucleic acid amplification products such as rolling circle amplification (RCA) and strand exchange amplification (SDA)^15^,^16^.

Unfortunately, it has been found further utilization of CHA in real-world detection rather than just proof-of-concept validations is still practically difficult and inflexible. A well-designed circuit may only functions efficiently at a narrow temperature zone, for example, ranging from 32 °C to 42 °C for a regular reaction optimized to perform at 37 °C^15^. Once the temperature fluctuates out, it requires laboriously shortening or extending sequence lengths according to temperature cooling or elevating^15^. We recently also realized CHA may be still inefficient or non-functional even after rational design. The main reason is most worthy-to-detect RNA or gene segments are actually containing highly self-folding structures that can block toehold binding and strand exchange steps involved in CHA pathway, finally inhibiting or ruining the whole reaction (Fig. 1B, column 2, Figure S1). The prevalence of such inhibition induced by unexpected “temperature fluctuation” or “self-folding” has perniciously impeded the broader applications of CHA and other nucleic acid circuits, especially in achieving point-of-care (POC) gene or RNA diagnostics.

Tracing back to the 60’s of last century, pioneers in nucleic acid research revealed amides, in particular formamide, could speed up DNA hybridization rate^17^ and meanwhile improve hybridization specificity^18^ as a hydrogen-bond destabilizer^19^. In the following several decades, these properties of formamide have been successively employed for increasing reaction rate or accuracy of many novel nucleic acid interactions^20^,^21^,^22^,^23^,^24^,^25^,^26^,^27^,^28^,^29^, including those milestones such as polymerase chain reaction^20^, sequencing^21^ and nanostructure assembly^26^,^27^,^29^. Inspired by these early findings, here we deeply explored the amide (especially formamide)-assistant mechanism in the CHA reaction and showed dramatic enhancement in the buffering ability of a CHA against unexpected self-folding or temperature-cooling. As a hybridization accelerator and moderate hydrogen bonder softener, formamide with an appropriate volume fraction (≤50%) could successfully revive a structure-inhibited CHA reaction, promote its catalytic rate by tens-to-hundreds times, and enlarge its functional temperature to much cooler than desired. Especially when organic amide was coupled with our self-engineered CHA system executing at as high as 45 °C to 60 °C (HT-CHA, Fig. 1B, column 3)^15^, the as-formed OHT-CHA can amplify efficient through 4 °C to 60 °C, exempt from rebuilding sequences or concerning target complexity (Fig. 1B, column 4). Such a flexible performance has never been achieved in previous reports. Detailed rules summarized in this article may motivate and enable many other advances in this field, both theoretically and practically. As a very important example (Fig. 1C), we employed OHT-CHA as a temperature-independent and structure-irrespective signal enhancer for loop mediated isothermal reaction (LAMP)^30^,^31^,^32^,^33^. LAMP is a most powerful point-of-care (POC) gene amplifier, but its direct detection has been analytically difficult due to structured products and frequent off-target reactions. OHT-CHA guaranteed a significant and reliable LAMP signal even in presence of down to 10^−19^ M virus genes of fetal Middle-East respiratory syndrome coronavirus (MERS-CoV) or Zaire Ebolavirus (Ebola). Moreover, one single set of OHT-CHA was qualified to any detection requirement, either in real-time at LAMP running temperature (~60 °C), or at end-point on a POC photon counter only holding environmental temperatures fluctuating between 4 °C to 42 °C. Gene detection based on LAMP thus turns ultra-reliable and environmental compatible, being ready in POC applications.

## Results

### Self-structures ruined a high temperature CHA (HT-CHA) at room temperature

**Figure 1:**
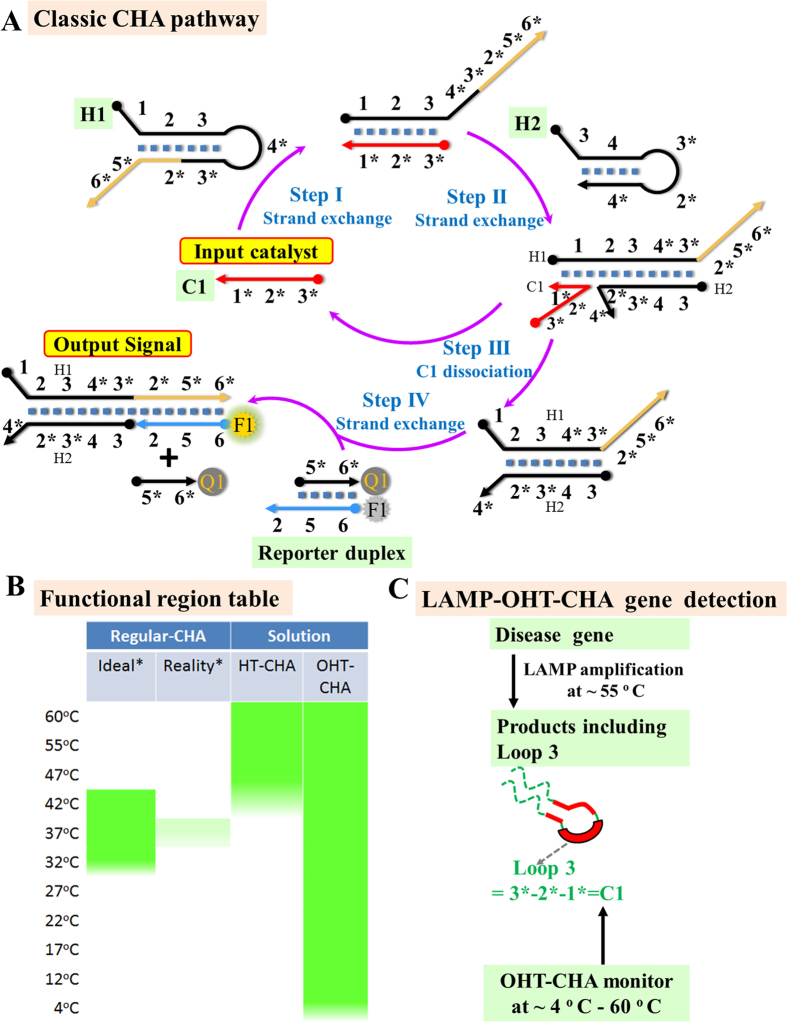
(**A**) Scheme of a classic four-step CHA pathway. Complementarity between numbered domains is denoted by an asterisk (*). (**B**) Schematic table showing average operating temperatures of an ideal regular CHA (Ideal), a structure ruined regular CHA (Reality), a high temperature CHA (HT-CHA), and an organic amide promoted HT-CHA (OHT-CHA). (**C**) Scheme of coupling OHT-CHA with LAMP to achieve ultra-sensitive, reliable and compatible gene diagnosis.

**Figure 2:**
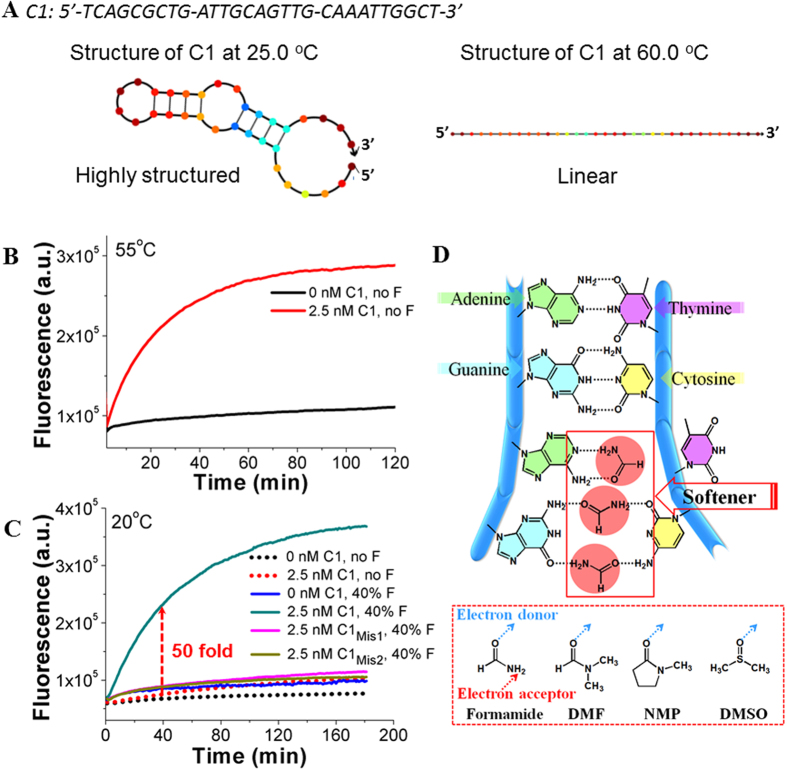
(**A**) The theoretically calculated secondary structures of C1 at 25 °C (Left) and 60 °C (Right), respectively. The calculation was made on open-access online software, NuPack^34^. The buffer condition was set to contain 200 mM NaCl and 5 mM MgCl_2_. (**B**) Fluorescent responses of HT-CHA at 55 °C with and without C1. (**C**) Fluorescent responses of HT-CHA (with no or 40% F) at 20 °C with and without C1. F represents formamide. C1mis1 and C1mis2 represent mismatched C1 with T-to-A and G-to-C single point mutation, respectively. (**D**) Illustration of proposed interaction between amide molecules and nucleobases. *Note*: (I) The bases occupied or base pairs weakened shown in Fig. 2D were randomly selected. The actual condition it could be any base or base pair. (II) Concentrations of all components, operating temperatures, inputs, amide concentrations, and instruments used for each Figure of this paper were also listed in Table S2 for convenient reading. Note: To meet different temperature requirements and prove the instrument-friendly property of our method, we used more than one fluorescent outputting instrument (Seeing Instruments section of supporting information). Therefore the data of y-axis of different figures may be in different scales.

Our demonstration started from specifically designing a HT-CHA for potential MERS-CoV virus detection. The reaction followed a classic four-step pathway^9^ shown in Fig. 1A and general rules summarized in our report of HT-CHA^15^. The catalyst (C1) sequence was designed as a selected 29-mer fragment within ORF1A region of MERS-CoV virus gene (MRES-1A, Fig. 2A)^31^,^33^. Customarily, we describe the DNA reaction components in terms of numbered domains, each of which contains 10–15 mer short sequence. Complementarity between numbered domains is denoted by an asterisk (*). In presence of C1, its domain 1 serves as a toehold to bind domain 1* on H1 and initiates a branch migration reaction to open the H1 stem (Fig. 1A, Step I). Domain 3 on H1 then serves as a second toehold to initiate another strand displacement reaction to open the H2 stem, forming the C1-H1-H2 intermediate (Fig. 1A, Step II). C1 can automatically dissociate from H1 and serve as a catalyst to trigger one other round of hairpin assembly reaction, leaving H1-H2 duplex as final product (Fig. 1A, Step III). Meanwhile, overhanging fragment (4*-3*-2*-5*-6*) on H1-H2 initiates one more toehold-mediated strand displacement that releases a fluorescence quencher (Q1) from a fluorescein amidite (FAM)-labeled oligonucleotide (F1), allowing the reaction process being monitored (Fig. 1A, Step IV).

As shown in Fig. 2B gotten at 55 °C, a sharp fluorescence increase was clearly observed when only 2.5 nM C1 was added into the CHA reaction, on contrast to a quasi-flat curve gotten from the reaction without any C1. Such high signal-background resolution was obtained through a temperature range from 47 °C to 60 °C (Figure S2), well meeting the high performance of a HT-CHA^15^. However, when the temperature was decreased to our room temperature fluctuating between 20 °C and 25 °C, the reaction with same amount of 2.5 nM C1 showed almost ignorable fluorescence increase during a whole 3 h monitoring period (Fig. 2C), which was more visually reflected by an almost 100-fold decrease in initial reaction rate (Δ relative fluorescent unit/Δ time_0–30min_, ΔRFU/Δt_0–30min_) compared with the one at 55 °C. According to our previous study^15^, it was predictable the HT-CHA could lose some catalytic efficiency at cooler temperature due to the slower dissociation of C1 from H1-H2-C1 complex (Fig. 1A, Step III). Here the completely loss in catalytic activity was more attributed to the undesirable secondary structures in C1 and fragment 4*-3*-2*-5*-6* formed at around 20 °C (Fig. 2A, left, and S3A). Calculation was made on open-access online software, Nupack, developed by Caltech University^34^. They seriously blocked the toehold binding and thus slowed down further branch migration step, finally ruining the whole reaction. This assumption could be confirmed by observing gradually recovered catalytic activities along with heating-up the reaction (Fig. 2B and S2). Upon temperature increase, these secondary structures would be gradually dissolved, and even eliminated when the temperature approached 60 °C (Fig. 2A, right, and S3B).

### Formamide significantly improved the buffering abilities of a CHA circuit against unexpected cooling and self-folding

When we started importing formamide (from 10% volume concentration) into the above reaction at room temperature, a formamide concentration-dependent recovery in catalytic rate (with 2.5 nM C1) was observed. In presence of 40% formamide, the catalytic rate reached as high as 50-fold compared to that without formamide (Fig. 2C and S4). Meanwhile, the non-catalytic reaction rate (without C1) still kept unchanged “zero” in the same recipe, about 500-fold less than that of catalytic reaction. Above surprising improvement could be partially explained by earlier finding^17^,^18^,^19^,^20^,^21^,^22^,^23^,^24^,^25^,^26^,^27^,^28^,^29^ and our experiments (Figure S5) that formamide could accelerate the hybridization rate. Being identically consistent with one other pioneering literature^22^, we also observed formamide could linearly decrease duplex melting temperature at a rate of approximate −6.5 °C/volume fraction (×100) formamide (Figure S6), suggesting formamide was meanwhile destabilizing the nucleobase pairs. In a mechanism derived from our and other’s understanding^22^, this phenomenon probably resulted from the special molecular size and composite of formamide. Carbonyl oxygen atom on formamide might serve as a hydrogen-bond competitor that directly occupies the position of one base, finally breaking-up a normal base pair. But meanwhile, formamide is also likely to just soften but not destroy a base pair through forming a base-formamide-base intermediate, in which both carbonyl oxygen atom and amino hydrogen atom take part in hydrogen bond formation. Formamide thus happened to gain similar function as heating and opportune force to unwind/soften those unexpected self-structures blocked the strand exchange reactions during the HT-CHA pathway. More intuitionally, formimade with appropriate amount was strong enough to melt those unexpected, relatively weaker self-folding structures in C1 and fragment 4*-3*-2*-5*-6*, but mild enough to remain the expected, stronger H1, H2, and H1-H2 duplexes. The softening effect also facilitated the process that C1 dissociated from H1-H2-C1 intermediate at low temperature. Overall, it was formamide’s synergistic function of both accelerating and destabilizing that greatly enhanced the fluidity of a suppressed HT-CHA at room temperature. A detection limit of 50 pM C1 was gotten at 25 °C (Figure S4), as sensitive as that gotten from a HT-CHA functioning at its desirable high temperature^15^.

**Figure 3:**
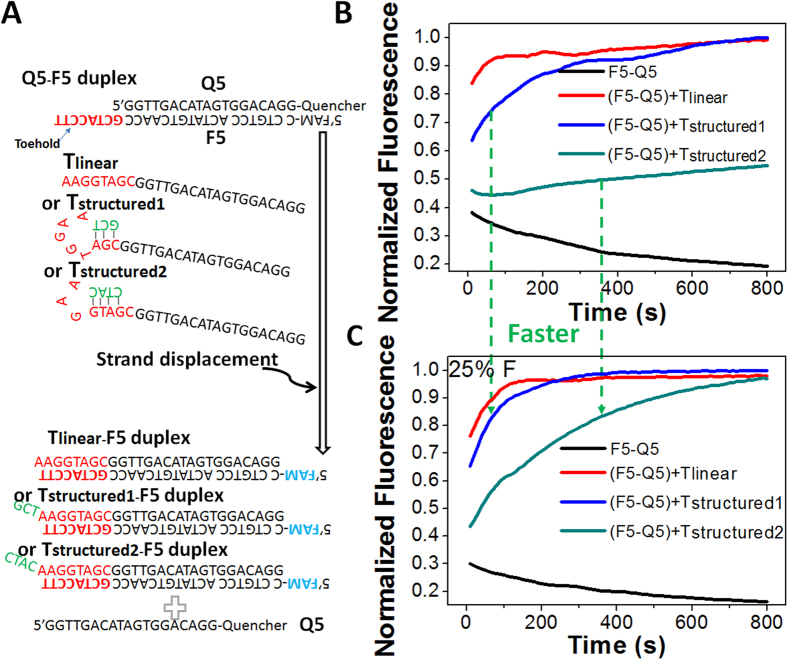
Demonstration that formamide can significantly accelerate toehold-mediated strand exchange reaction inhibited by small scale self-folding structures. (**A**) Scheme of toehold mediated strand exchange reaction triggered by T_linear_, T_structured1_, and T_structured2_, respectively. The three triggers hold the same linear sequence of T_linear_ that could displace Q5 away from F5. The segment in red was toehold binding domain, which was partly blocked in T_structured1_ and T_structured2_ at different degrees. After strand exchange, fluorescence of FAM on F5 was quenched by the Quencher on Q5. (**B**) and (**C**) Real-time fluorescent kinetic monitoring right after T_linear_, T_structured1_, or T_structured2_ was added into F5-Q5 duplex solution without (**B**) and with (**C**) 25% formamide. The experiments were carried out at 25 °C.

**Figure 4:**
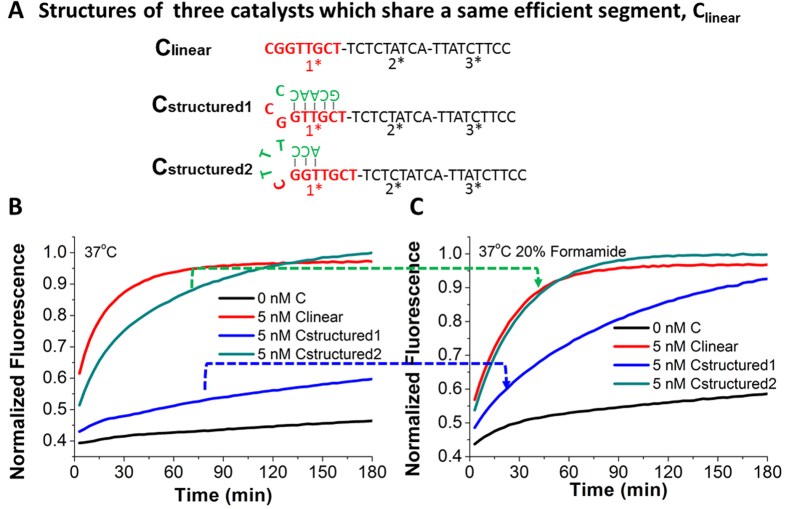
Demonstration that formamide can significantly accelerate a 37 °C regular CHA reaction inhibited by small-scale self-folding structures. (**A**) Sequences and structures of three catalysts, C_linear_, C_structured1_, and C_structured2_, respectively. The three catalysts hold the same C_linear_ sequence that can trigger the CHA reaction at 37 °C. The segment in red was the toehold binding domain, which was partly blocked in C_structured1_ and C_structured2_ at different degrees. (**B**) Real-time fluorescent kinetic monitoring right after buffer, C_linear_, C_structured1_, or C_structured2_ was added into CHA components without (**B**) and with (**C**) 20% formamide.

Summarized from our systematic discussion in both main text (Figs 3 and 4) and supporting information (Figure S5–S9), it could be concluded that the formamide-enhanced structure-buffering and thermo-buffering abilities could be generalized to any one step strand exchange reaction (Fig. 3) and CHA circuit (Fig. 4), no matter it was a HT-CHA (Fig. 2C) or regular CHA that was ought to proceed at around 37 °C (Fig. 4). Just through slightly adjusting the formamide content, we could more or less revive a CHA reaction from self-folding-lagged passivation, and at the time widen its efficient region to cooler circumstances than desired functional temperature (Fig. 2C and S9). However, as a hydrogen-bond softener, the effect of formimade on an ideal (no self-folding) strand exchange or CHA reaction would not be definitely positive at desired functional temperatures or above, but highly depends on the toehold length (or sequence) of each strand exchange step. Notably, the advantage of using formamide in a circuit would be especially dramatic, much more obvious compared with in a one-step strand exchange reaction or simple hybridization. The reason was a nucleic acid circuit was an integration of multiple strand exchange reactions. Therefore, the promotional effect of formamide on each strand exchange step would be correspondingly integrated and accumulated in a circuit.

### Establishment of organic amide promoted HT-CHA system (OHT-CHA)

Even though the promotional effect of formamide on CHA was general, using formamide together with a thermo-stable one (HT-CHA) could endow a circuit maximum thermo- buffering ability and thus was especially encouraged. As confirmed (Figure S10), in assistance of formamide, a HT-CHA could function efficiently through above 4 °C to 60 °C, without concerning target complexity or rebuilding component sequences. To emphasize this unprecedented and remarkable advance, such combination of organic amide with HT-CHA was in general named OHT-CHA. The above HT-CHA and OHT-CHA using C1 as catalyst were then specified as HT-CHA1 and OHT-CHA1, respectively. From observing the optimized formamide concentration in OHT-CHA1 at different temperatures (Figure S10), the highest necessity of using formamide sit at temperatures below 47 °C. The more the temperature was lower than 47 °C, the more formamide was required to achieve satisfied promotion. It should be noted that the optimized amount and necessary temperature of formamide have to be experimentally determined, since it might vary a lot for different CHA sets, and still be difficult to be theoretically calculated. But at any situation formamide should be controlled less than 50%. Otherwise over amounted formamide would seriously destabilize expected H1, H2 and reporter duplexes as well. Some other amides, such as dimethyl sulfoxide (DMSO), dimethyl formamide (DMF), and N-methyl-2-pyrrolidone (NMP), were also found with similarly effect to formamide (Figure S11). Nevertheless, under equal amount formamide provided the highest signal-to-background ratio for OHT-CHA1. Such high performance of formamide further showed its moderate strength in softening hydrogen bond, and thus remained its usage in all of our later experiments.

### Significant improvement of OHT-CHA in the single mismatch discrimination at low temperature

Other than promoting catalytic reaction rate and providing wider thermo-buffering ability, amide could also significantly increase detection selectivity of a circuit at low temperature. As shown in Fig. 2C, only a single mutation at the middle of C1 toehold domain (e.g. C1mis1) or at starting-point of branch migration (e.g. C1mis2) was able to inhibit catalytic reaction to almost undetectable, about 120-fold decrease in initial reaction rate, which was even more than 10 times higher than that of either one step strand exchange^35^,^36^ or a regular 37 °C CHA^9^.

### Further demonstration of generality of the OHT-CHA

**Figure 5:**
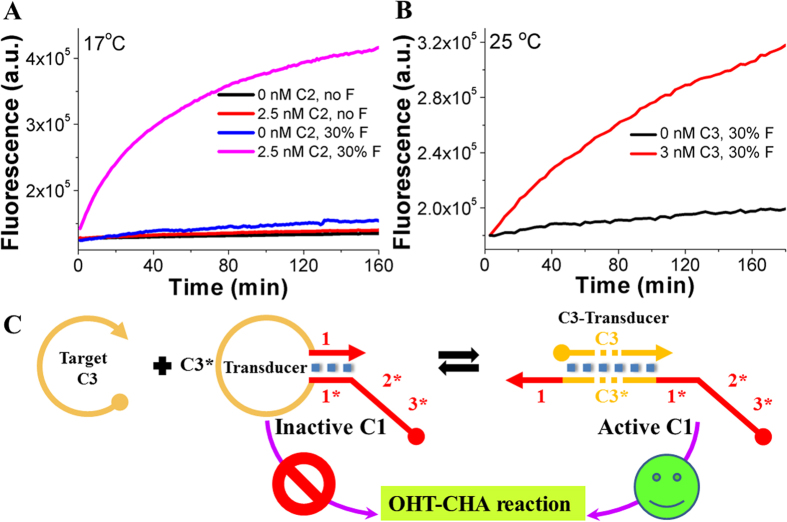
Generality of OHT-CHA. (**A**) Fluorescent responses of quite another OHT-CHA (OHT-CHA2) at 17 °C, with and without C2. (**B**) Fluorescent responses of OHT-CHA1 triggered by 0 nM and 3 nM C1-non-relevant target, C3, in presence of a hairpin transducer. The pathway for this strategy was shown in Fig. 5C. The explanation for this pathway was in caption of Figure S13A.

The generality of OHT-CHA could be proved in two simple ways. First, we applied the similar OHT-CHA strategy into one other HT-CHA designed with completely different sequences (C2, H3, H4, F2, Q2). In presence of formamide (30%), the new OHT-CHA (OHT-CHA2) again showed a significant performance surge at room temperature of 17 °C (Fig. 5A, 55 °C shown in Figure S12). Second, as shown in Figure S13, 5B and 5C, using one other target (C3 here) to open a hairpin transducer in which toehold domain of C1 was previously blocked by the stem of transducer, the unlocked C1 could then trigger the OHT-CHA1 (H1, H2, F1 and Q1) efficiently at 25 °C. This success further generalized OHT-CHA to those sequences non-relevant to its components.

### Application of OHT-CHA in improving reliability and environment-compatibility for real-world virus diagnosis

**Figure 6:**
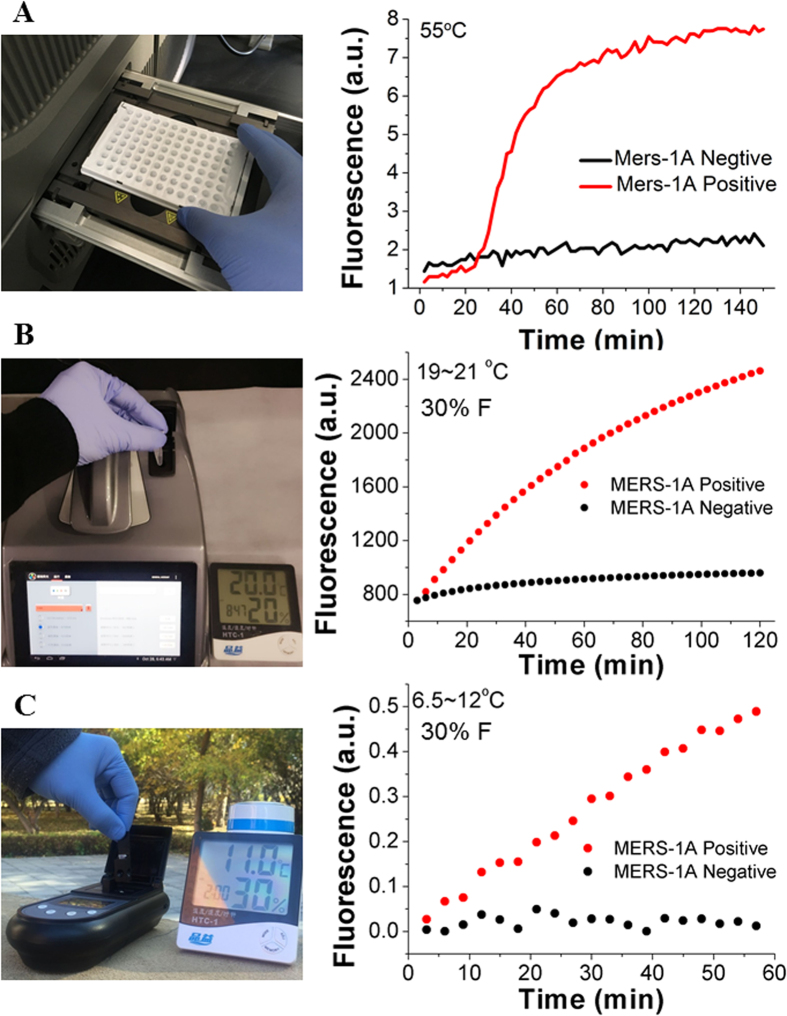
Demonstration that LAMP-OHT-CHA combination is environmental ultra-compatible. Fluorescent responses of OHT-CHA1 to LAMP products amplified from synthetic MERS-1A DNA negative (0 copy) and positive (2000 copies, 6 × 10^−17^ M) samples. Data were collected, respectively, on (**A**) a professional real-time PCR system at 55 °C simultaneously with LAMP reaction, (**B**) a fluorescence (FL) spectrum under room temperature fluctuating from 19 °C to 21 °C, and (**C**) a portable FL detector under outdoor temperature fluctuating from 6.5 °C to 12 °C. For Fig. B and C, Data were at end-point collected after LAMP products were manually added into CHA reaction. Other useful notes about OHT-CHA-LAMP detection see the last page of supporting information).

**Figure 7:**
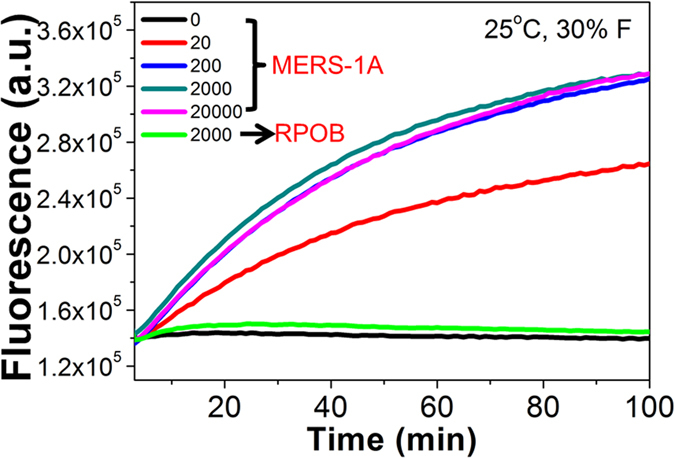
Demonstration that LAMP-OHT-CHA combination is ultra-sensitive and selective. Fluorescent responses of OHT-CHA1 (with 30% formamide) to LAMP products amplified from different copies of MERS-1A DNA samples. The green curve was gotten from using LAMP product of M. tuberculosis rpoB gene (RPOB) segment as an anti-interference control to trigger OHT-CHA1. LAMP reaction was carried out for 20 min at 60 °C. The fluorescent reading was performed at 25 °C.

To further verify the application of OHT-CHA in monitoring trace amount real-world target much less than pM level, LAMP was innovatively imported here as an assistant sensitivity booster. LAMP is a most point-of-care promising isothermal gene amplifier^32^ but has been underused due to its complex products and haunted non-specific false amplicons (Figure S14)^37^,^38^. Our recent advances have proven one step strand exchange reaction (OSD) could solve this problem by specifically probing single strand loop amplicons among LAMP products^30^,^31^,^33^. Here displacing OSD with CHA circuit may provide further signal enhancement upon anti-false function (Fig. 1C and S13B). In detail, the 29-mer MERS-1A gene segment, with the same sequence as C1, was designed to be one of the loop sequences (Loop 3 in Figure S14) among LAMP amplicons amplified from MERS-1A. Simultaneously with or after MERS-1A-posivie LAMP reaction, the accumulated Loop 3 (C1) would initial OHT-CHA1 (without formamide) at 55 °C, either in real-time (Fig. 6A) or at end-point (Figure S15). Besides performing the detection at 55 °C on a professional real-time PCR machine (Fig. 6A, S15), addition of formamide now also allowed the same OHT-CHA1 executing end-point detection on fluorescence (FL) facilities with no temperature control (Figure S16, 6B and 6C). For example, it was very excited to see OHT-CHA1 behaved the same well on a common FL spectrum (under room temperature fluctuating from 19 °C to 21 °C, Fig. 6B), and a portable FL photon counter with resource-poor settings (under outdoor temperature fluctuating from 6.5 °C to 12 °C, Fig. 6C). Control experiments indicated even at such low temperature the detection was still so specific and sensitive (Fig. 7). After as short as 20–30 min LAMP reaction, OHT-CHA1 could provide accurate “YES”-or-“NO” detection for as low as 20 copy MERS-1A (≈6 × 10^−19^ M, or 600 zM, Fig. 7), well meeting the practical requirement for MERS-CoV virus detection^31^,^33^. Meanwhile, the special second-order kinetic curve avoided the misreading brought by easy-shifted fluorescence intensity generated in regular end-point fluorescence assay. Practically, the negative control reading could be even omitted. Note here while ultra-sensitivity could be realized, there hardly was dose-discrimination. First, as might have been expected for a robust amplification reaction such as LAMP, primers in reactions with different amount templates have been all consumed in a short given time, producing similar amounts of amplicons. Second, similar to other powerful isothermal amplifications, LAMP with different samples might randomly produce unrepeatable side amplicons, which could affect the accurate quantification of correct amplicons.

**Figure 8:**
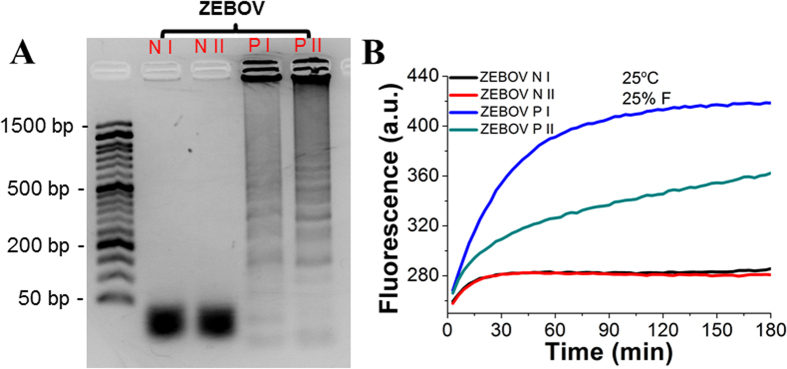
Demonstration that LAMP-OHT-CHA combination is ultra-reliable. (**A**) Electrophoresis and (**B**) Characterization with OHT-CHA3 of LAMP amplicons from synthetic ZEBOV DNA negative (0 copy, NI and its parallel, NII) and positive 2000 copies (6 × 10^−17^ M, PI and its parallel, PII) samples. Curves in Fig. B were collected on a portable real-time PCR system at room temperature of 25 ^o^C.

We also tried to use OHT-CHA to detect LAMP amplicons of Zaire Ebolavirus synthetic DNA templates (ZEBOV)^33^. Just as shown in the gel electrophoresis (Fig. 8A), LAMP products amplified from parallel ZEBOV positive samples (PI and PII) run into different patterns and it was hard to get clear diagnostics. Now such “puzzle” could be easily solved by using ZEBOV targeting OHT-CHA (OHT-CHA3). Products from both PI and PII could generate OHT-CHA fluorescence curves, verifying both samples contained correct amplicons and were ZEBOV positive (Fig. 8B). While the slower slope with PII indicated the correct amplicons were diluted by more side non-specific products compared with that with PI. These experiments further confirmed CHA’s amplifiable necessity that guaranteed a high and reliable signal in response to seriously diluted amplicons. But it should be notable due to LAMP reactions, especially being accompanied with side reactions, could hardly be completely reproduced from time to time. The gel pictures and kinetic curves in Fig. 8 could show different patterns and slopes whenever the experiments are to be repeated.

## Discussion

In this paper, we presented the first systematic demonstration showing how formamide and other amide denaturants were so deeply and helpfully involved in the CHA reactions. Through step-by-step study we reproduced formamide’s critical function as an efficient hybridization accelerator, and confirmed its destructiveness as denaturant was particularly moderate, compared with other amides. Within a wide concentration range, formamide was strong enough to smooth those unexpected, small-scale structures, but weak enough to remain most expected ~20-mer duplexes generally used in strand exchange reactions, especially at ambient environmental temperatures. Such a synergistic effect of acceleration and mild smoothing finally made formamide dramatically effective to revive a structure-inhibited strand-exchange. This superiority was even enlarged when multiple strand exchange reactions were integrated into a nucleic acid CHA circuit. In complicated medium such as serum (e.g. 50% fetal bovine serum, Figure S17), formamide was found functioned as well as, and even better than in pure buffer condition. Besides reviving the structure-inhibited CHA reaction, formamide could also effectively inhibit those enzymes in serum that could degrade CHA components. Especially when formamide was coupled with a HT-CHA, the as formed OHT-CHA has showed significant improvement for gene detection in all aspects of signal amplitude, specificity, and environment-comparability. We have tried to use different types of fluorescence readers to monitor the LAMP-OHT-CHA combination. Even through different instruments have different temperature controlling abilities, different data collecting and processing accuracies, and different POC comparability, the OHT-CHA could perform equally well on these various instruments, providing its beautiful climbing curve only in presence of the correct virus genes. It should be noted that in this paper, we only used easily-prepared and contamination-free synthetic DNA templates but not real virus RNA because our recent study have already demonstrated the amplification and anti-interference ability of reverse transcription LAMP (RT-LAMP) for both ZEBOV and MERS RNAs were equally efficient to that of LAMP for MERS-1A and ZEBOV DNA templates)^30^,^31^,^33^. And formamide was not encouraged being added into the real-time detection at as high as ~60 °C. It may do harm to the DNA polymerase and delay the LAMP reaction. To avoid contamination generated during any open-tube step after LAMP reaction, transporting OHT-CHA components through a sealed flow set-up was suggested.

## Conclusion

In conclusion, we have shown the introduction of formamide to smooth the undesired structures in strand exchange and CHA reactions, which for the first time allowed us to extend a single CHA functioning at flexible operation temperatures ranging from right above ice point to 60 °C. From the view of point-of-care, we can see that the as-named OHT-CHA may boost the usage of nucleic circuitry in detecting diseases at somewhere there is poor temperature control. Such possibility was well demonstrated when we realized ultra-sensitive and-specific detection of MERS-1A and ZEBOV in various environments. This interesting advance has also deepened our insight of strand exchange, nucleic acid circuitry and their relationship with underused organic hybridization accelerators. Its applications are supposed to be generally extended to other strand exchange based circuits, making them much easier to design and more powerful. Being encouraged by such a remarkable promoting function of amide organic, other types of chemicals^17^,^18^,^19^,^20^,^21^,^22^,^23^,^24^,^25^,^26^,^27^,^28^,^29^ holding similar or even better effect are highly deserved further exploration, comparison and theoretical calculation.

## Methods

### Materials and instruments

Instruments and materials (including buffers and chemicals) were listed in supporting information. Sequences and reaction conditions for each figure were listed in Table S1 and Table S2, respectively.

### Real-time HT-CHA or OHT-CHA fluorescence kinetic reading

All the hairpins and reporters were annealed at 95 °C for 5 min and cooled down to 25 °C at a rate of 0.1 °C/s before use. Unless otherwise indicated, all HT-CHA’s kinetic reading was performed in 1× IsoMg buffer. All OHT-CHA’s kinetic reading was performed in 1× IsoMg buffer in presence of different volume concentrations of amide solvent. In both circumstances final CHA components in a reaction mixture contained 50 nM H1 (or H3, or H5), 200 nM H2 (or H4, or H6), and 50 nM Reporter duplex with and without catalyst. In Reporter duplex, [F1 (or F2, or F3)] was 50 nM, 1/2 that of [Q1 (Q2, or Q3)]. The fluorescence signal of 17 μL each CHA mixture was recorded every 1 to 3 min at different temperatures on different instruments. *NOTE:* Concentrations of CHA components, operating temperatures, inputs, amide concentrations, and instruments used for each Figure of this paper were also listed in Table S2 for convenient reading.

### Cloning of MERS-CoV and Zaire Ebolavirus VP30 gBlocks and PCR amplification of transcription templates (Making MERS-1A and ZEBOV DNA templates)

Synthetic MERS-1A and ZEBOV templates were obtained with the same protocol as ref. 32. Briefly, all DNA polymerase chain reaction (PCR) amplification reactions were performed using high-fidelity Phusion DNA polymerase (NEB), according to the manufacturer’s instructions. The gBlock double stranded DNA surrogates of MERS-CoV and ZEBOV genetic loci were designed to include a T7 RNA polymerase promoter at their 5′-ends to enable subsequent transcription. These gBlocks were cloned into the pCR2.1-TOPO vector (Life Technologies, Carlsbad, CA, USA) by Gibson assembly using the 2× mastermix (NEB) according to the manufacturer’s instructions. Cloned plasmids were selected and maintained in an *E. coli* Top10 strain. Plasmid minipreps were prepared from these strains using the Qiagen miniprep kit (Qiagen, Valencia, CA, U.S.A). All gBlock inserts were verified by sequencing at the Institute of Cellular and Molecular Biology Core DNA Sequencing Facility.

For performing *in vitro* run-off transcription, transcription templates cloned in a pCR2.1-TOPO vector were amplified from sequenced plasmids by PCR using Phusion DNA polymerase. Final PCR products (MERS-1A and ZEBOV) were verified by agarose gel electrophoresis and then purified using the Wizard SV gel and PCR Clean-up system, according to the manufacturer’s instructions (Promega, Madison, WI, USA).

Synthetic *M. tuberculosis rpoB* gene (RPOB) segment was generated from commercial genomic DNA of the virulent strain H37Rv (ATCC, Manassas, VA, USA) in the same protocol described above.

### Standard LAMP reaction

Mixtures containing different copies of template (MERS-1A or ZEBOV), 1 μM each B1c-B2 and F1c-F2, 0.25 μM each B3 and F3, 0.5 μM LP (no LP in ZEBOV LAMP), 1 M betaine, and 0.4 mM dNTPs in a total volume of 24 μL 1 × IsoMg buffer were heated to 95 °C for 5 to 10 min, followed by chilling on ice for 2 min (*NOTE*: This pre-denaturing process was not absolutely necessary but resulted in improved sensitivity). Then, 1.5 μL (8 U) of Bst DNA polymerase 2.0 was added to initiate the LAMP reaction. Unless otherwise indicated, the reactions (with a final volume of 25 μL) were incubated for 1.5 hour in a thermal cycler maintained at ~55 °C–60 °C. To characterize LAMP reaction, a 5 μl aliquot of the reaction mixed with 2 μl Gel-Dye Super Buffer Mix was then analyzed by electrophoresis through a 1% agarose gel. Gel analysis of LAMP products was performed in a fume-cupboard completely separate from the normal laboratory space. This precaution was taken to minimize the spread of LAMP amplicon contamination. (*Note*: The LAMP reaction volume could be at least increased to 100 μL without losing sensitivity.)

### Standard real-time and end-point LAMP-HT-CHA and LAMP-OHT-CHA detection

#### End-point

A total 17 μL liquid containing certain volume of formamide, certain volume of 10×Iso buffer, certain volume of 100 mM MgCl_2_ and 3 μL concentrated CHA component mixture (annealed two hairpins and Reporter duplex) were added into every 25 μL standard LAMP products, followed by immediate fluorescent kinetic reading. Unless otherwise indicated, in a final LAMP-CHA mixture there were different volume concentrations of formamide solvent (usually 25% or 30%) and CHA components of 50 nM H1, 200 nM H2, and 50 nM Reporter duplex. In Reporter duplex, [F1 (or F2, or F3)] was 50 nM, 1/2 of [Q1 (Q2, or Q3)]. The buffer component was same as 1× IsoMg buffer. For experiments that generated Figure S16 and 6C, CHA components of 200 nM H1, 200 nM H2, and 200 nM Reporter duplex were used instead. In Reporter duplex, [F1 (or F2, or F3)] was 200 nM, equal to [Q1 (Q2, or Q3)]. *NOTE:* To avoid contamination generated during any open-tube step after LAMP reaction, transporting OHT-CHA components through a sealed flow set-up was suggested.

In fluorescent reading, the fluorescence signal of 17 μL each final reaction mixture was recorded every 1 to 3 min on a LightCycler^®^ 96 Real-Time PCR System (Figure S15) or a COYOTE Mini-8 Portable Real-time PCR system (Fig. 8). Fluorescence reading on DeNovix DS-11 + FX spectrophotometer (Fig. 6B) and HG-2 Portable Fluorescent Detector (Fig. 6C) used a 42 μL final reaction mixture instead of 17 μL. *NOTE*: Due to DeNovix DS-11 + FX spectrophotometer and HG-2 Portable Fluorescent Detector were lack of multi-well reading function, the kinetic data points for each sample were manually collected separately.

#### Real-time

A 25 μL standard LAMP reaction in presence of 2000 copies MERS-1A was carried and monitored at 55 °C every 1 to 3 min with LightCycler^®^ 96 Real-Time PCR System (Fig. 6A) except that 200 nM H1, 200 nM H2, and 200 nM Reporter duplex were simultaneously included. *NOTE*: Here the 3′end of H1, H2, and F1 were protected with invert-dT to avoid unexpected enzymatic extension.

## Additional Information

**How to cite this article**: Zhu, Z. *et al*. Strand-Exchange Nucleic Acid Circuitry with Enhanced Thermo-and Structure- Buffering Abilities Turns Gene Diagnostics Ultra-Reliable and Environmental Compatible. *Sci. Rep.* **6**, 36605; doi: 10.1038/srep36605 (2016).

**Publisher’s note:** Springer Nature remains neutral with regard to jurisdictional claims in published maps and institutional affiliations.

## Supplementary Material

Supplementary Information
