# Supplementary material for: Strand-Exchange Nucleic Acid Circuitry with Enhanced Thermo-and Structure- Buffering Abilities Turns Gene Diagnostics Ultra-Reliable and Environmental Compatible
Source: Sci Rep. 2016 Nov 4;6:36605. doi: 10.1038/srep36605 (PMC5095676; doi:10.1038/srep36605)
Supplement: Supplementary Information [file srep36605-s1.doc]

**Supporting Information for**

**Strand-Exchange Nucleic Acid Circuitry with Enhanced Thermo-and Structure- Buffering Abilities Turns Gene Diagnostics Ultra-Reliable and Environmental Compatible**

Zhentong Zhu^1,2‡^, Yidan Tang^1,2‡^, Yu Sherry Jiang^3^, Sanchita Bhadra^3^, Yan Du^3^, Andrew D. Ellington^3^ and Bingling Li^1,2*^

1. State Key Lab of Electroanalytical Chemistry, Changchun Institute of Applied Chemistry, Chinese Academy of Science, Changchun, Jilin, 130022, P.R. China.

2. University of Chinese Academy of Sciences, Beijing, 100039, China

3. Center for Systems and Synthetic Biology, University of Texas at Austin, Austin, Texas 78712, United States.

**Corresponding Author**

Email: [Binglingli@ciac.ac.cn](mailto:Binglingli@ciac.ac.cn)

**Author Contributions**

‡These authors contributed equally.

**1. Experiment section**

**Materials**. All chemicals were of analytical grade and were purchased from Sangon Biotech (Shanghai, China) unless otherwise indicated. Dimethyl formamide (DMF) were obtained from Beijing Chemical works (Beijing, China). Formamide, dimethyl sulfoxide (DMSO), and N-methyl-2-pyrrolidone (NMP) were obtained from Xilong Chemical Co., Ltd. (Guangdong, China). Fetal bovine serum was obtained from Zhejiang Tianhang Biotechnology Co.,Ltd. (Hangzhou, China). All oligonucleotides were ordered from Sangon Biotech (Shanghai, China). Oligonucleotide sequences are summarized in **Table S1**. All oligonucleotides were stored in H_2_O or 1×TE (pH 7.5) at -20 °C. Bst 2.0 DNA polymerase and 10 × Isothermal buffer (10 × Iso) was obtained from New England Biolabs (Ipswich, MA, U.S.A.). Buffers used here were:

- 1×TNaK (20 mM Tris-HCl, 140 mM NaCl, 5 mM KCl, pH 7.5)
- 1× Iso (20 mM Tris-HCl, 10 mM (NH_4_)_2_SO_4_, 50 mM KCl, 4 mM MgSO_4_, 0.1% Tween 20, pH 8.8)
- 1× IsoMg (1× Iso Buffer added with 1 μM dT21 and 2 mM Mg^2+^, finally 1 μM dT21, 20 mM Tris-HCl, 10 mM (NH_4_)_2_SO_4_, 50 mM KCl, 4 mM MgSO_4_, 0.1% Tween 20, pH 8.8)

**Instruments**. The concentrations of the DNA suspensions were measured by UV spectrophotometry using the DeNovix DS-11+ FX spectrophotometer (DeNovix Inc., Wilmington, DE, USA). Agarose gel electrophoresis was driven by JY600C Universal Power Supply (JUNYI DONGFANG Electrophoresis Co., Ltd., Beijing, China) and imaged by JY04S-3C Gel Document Imaging System from the same company. To meet different temperature requirements and prove the instrument-friendly property of our method, we used more than one fluorescent outputting instruments, including 1) Applied Biosystems StepOnePlus™ Real-Time PCR System (Thermo Scientific, Wilmington, DE, U.S.A., for **Figure 2**, **Figure 3-5**, **Figure 6A**, **Figure 7**, **Figure S1**, **Figure S2**, **Figure S4-S12**, **Figure S16**. 2) LightCycler® 96 Real-Time PCR System (Roche Life Science , NC, U.S.A., for **Figure S15**); 3) DeNovix DS-11+ FX spectrophotometer (DeNovix Inc., Wilmington, DE, USA, for **Figure 6B**); 4) HG-2 Portable Fluorescent Detector (Huguo Scientific Instrument Co., Ltd., Shanghai, China, for **Figure 6C**); and 5) COYOTE Mini-8 Portable Real-time PCR system (Coyote Bioscience, Inc., Beijing, China, for **Figure 8** and **Figure S17**). Except DeNovix DS-11+ FX spectrophotometer and HG-2 Portable Fluorescent Detector, all fluorescent instruments support plate reading that allows automatically kinetic reading of 8 to 96 samples at the same time. Oppositely, data points on DeNovix DS-11+ FX spectrophotometer and HG-2 Portable Fluorescent Detector for each sample have to be manually collected separately.

**2. Supporting Figures and Tables**

**
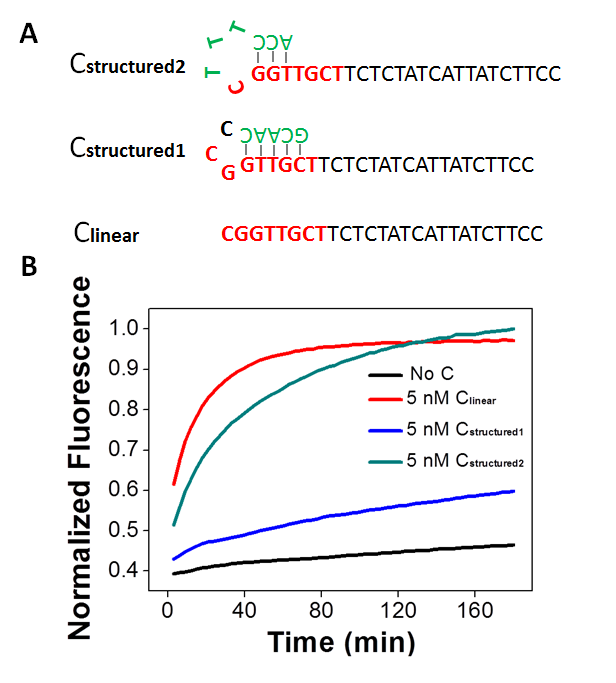
**

**Figure S1. Demonstration that self-folding structures can sharply inhibit the catalytic rate of a regular CHA reaction functioning at 37 ^o^C.** A) List of three catalysts with and without self-folding structures in the toehold binding domain. The three catalysts hold the same “active catalytic sequence” of “CGGTTGCTTCTCTATCATTATCTTCC” that was designed to trigger a regular CHA reaction at 37 ^o^C. The bases in red represent the toehold domain of the catalyst. B) Florescence responses of a regular CHA at 37 ^o^C in 1×TNaK buffer, in presence of to 0 nM (black) any catalyst, 2.5 nM C_linear_ (red), 2.5 nM C_structured1_(blue), and 2.5 nM C_structured2_ (green), respectively. ***NOTE:*** *Concentrations of CHA components, operating temperatures, inputs, amide concentrations, and instruments used for each Figure of this paper were also listed in* ***Table S2*** *for convenient reading*.

**
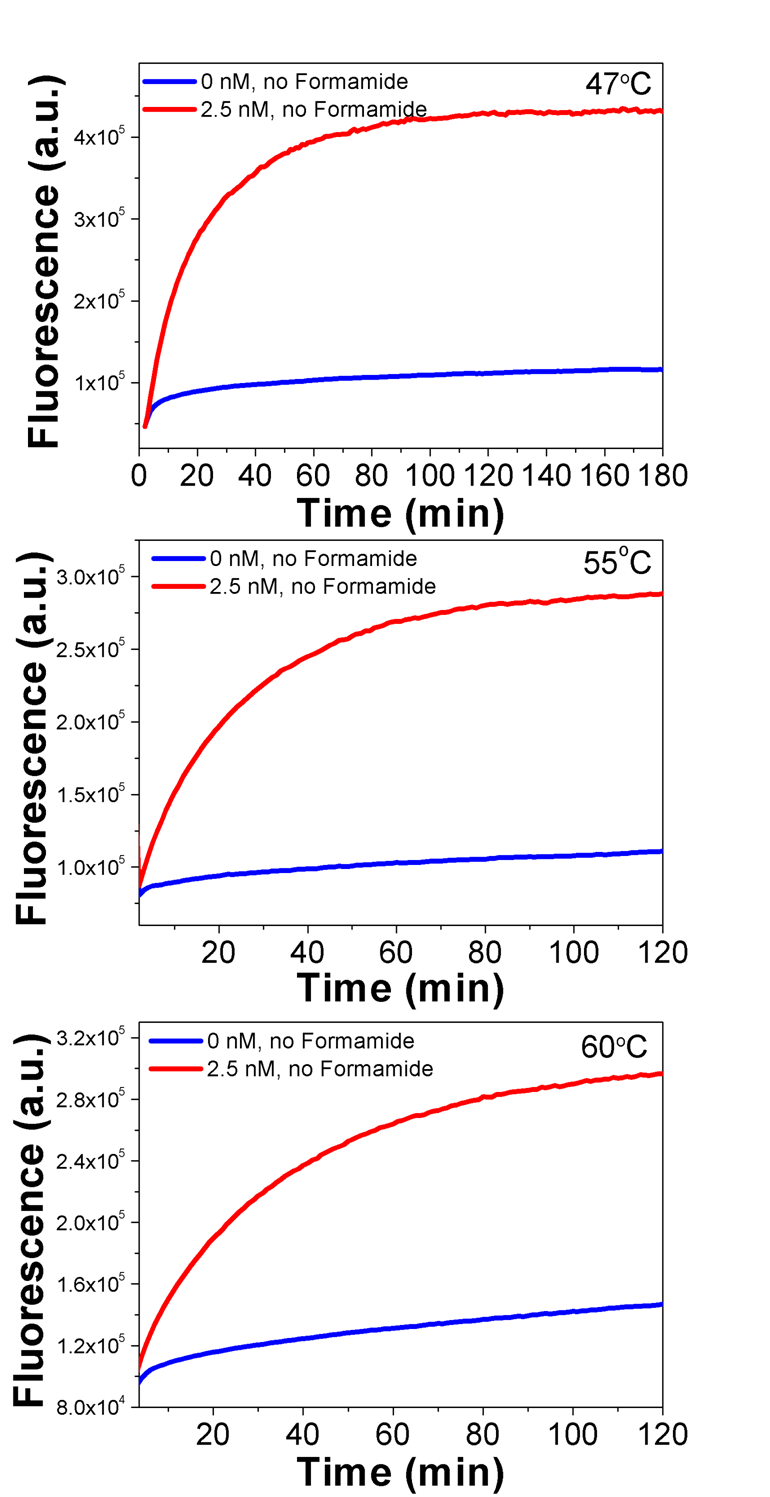
**

**Figure S2.** Fluorescence responses of HT-CHA (HT-CHA1) at 47^o^C-60^o^C without formamide, in presence of to 0 nM (blue) and 2.5 nM (red) C1. The high signal-background resolution was consistent with our previous research^S1^.

**
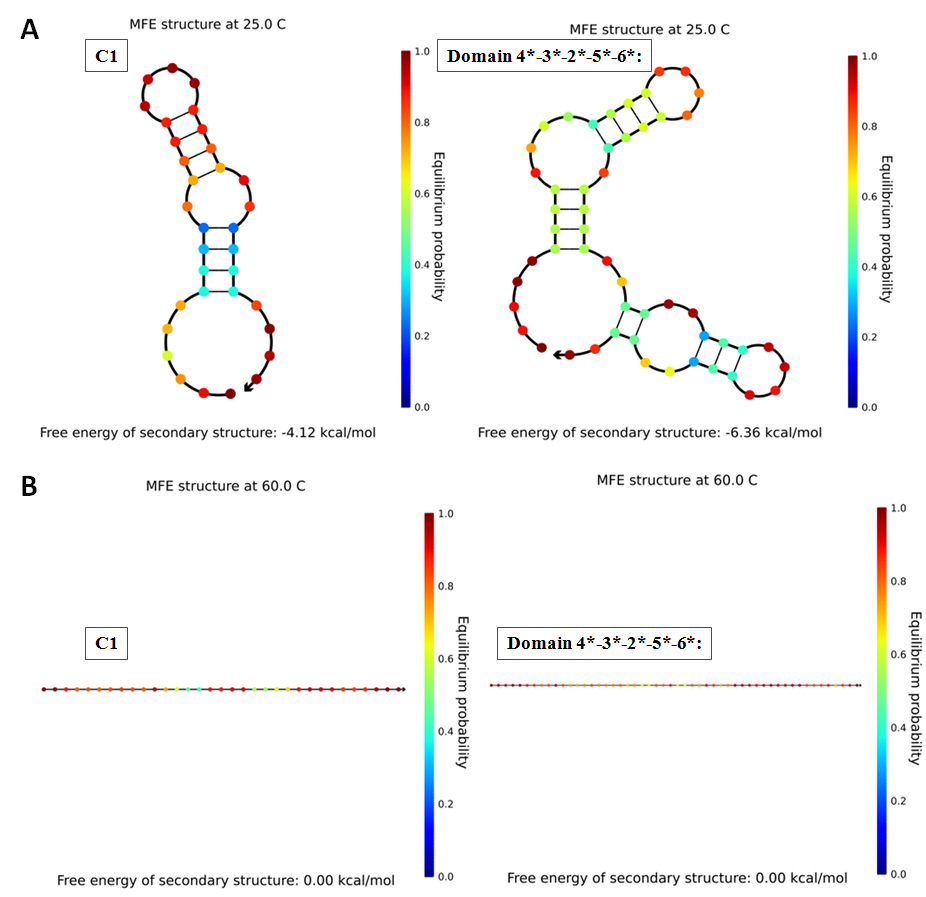
**

**Figure S3.** The theoretically calculated secondary structures of C1 (left) and segment 4*-3*-2*-5*-6* within H1 (right) at 25^o^C (A) and 60^o^C (B), respectively. The calculation was made on open-access online *Nupack* software developed by Caltech University. The buffer condition was set to contain 200 mM NaCl and 5 mM MgCl_2_.


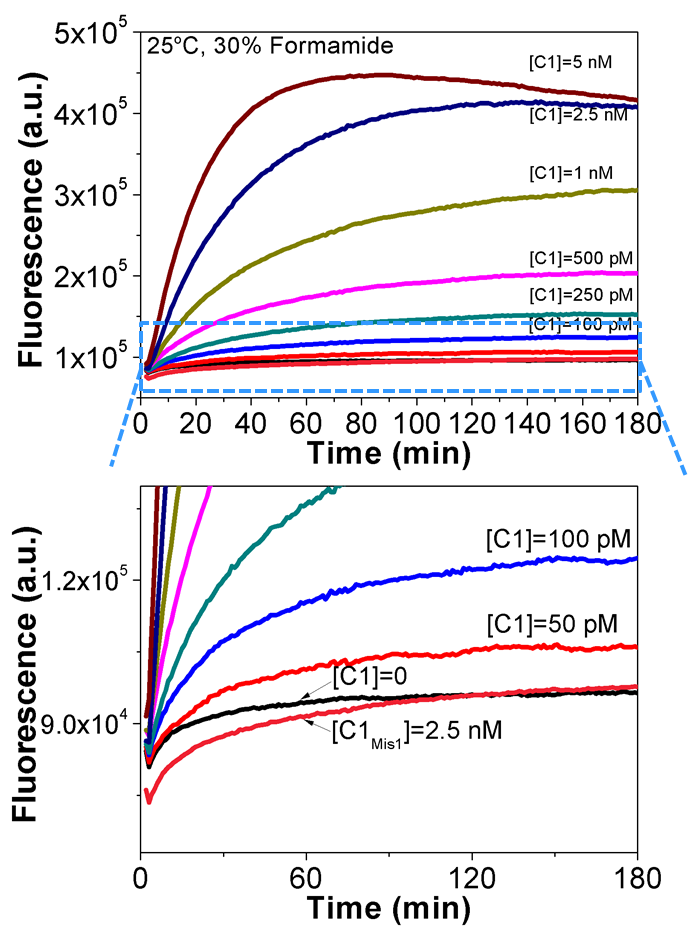


**Figure S4**. Fluorescent responses of OHT-CHA (OHT-CHA1) containing 30% formamide at 25 ^o^C, in presence of different concentrations of C1 and 2.5 nM C1mis1. Lower figure showed curves enlarged from those no discriminable ones in upper figure. For this figure initial reaction rate with 50 pM C1 would be already discriminated from that with either non-C1 background or 2.5 nM C1mis1.

- **Systematic discussion of formamide’s function in promoting CHA**

**Summarized** from our systematic discussion in both main text and supporting information below, it can be concluded that the formamide-enhanced structure-buffering and thermo-buffering abilities can be generalized to any CHA circuit, no matter it is a HT-CHA or regular CHA that is ought to proceed at around 37 ^o^C. Just through slightly adjusting the formamide content, we can more or less revive a CHA reaction from self-folding-lagged passivation, and at the time widen its efficient region to cooler circumstances than desired functional temperature. However, as a hydrogen-bond softener, the effect of formimade on an ideal (no self-folding) strand exchange or CHA reaction would not be definitely positive at desired functional temperatures or above, but highly depends on the toehold length (or sequence) of each strand exchange step.

Step-by-step discussion was listed as following **Figure S5-S9**.

1. **Formamide accelerates nucleic acid hybridization.**

As shown in **Figure S5B**, the slope of kinetic curve during the initial hybridization period is sharper and sharper along with formamide volume concentration is increasing. It demonstrates formamide is accelerating the fluorescence quenching process, more precisely, the hybridization process of F4 and Q4. This phenomenon is consistent with earlier report^S2^ and could be explained through formamide’s function that provides DNA an enriched microenvironment that can increase DNA local concentrations.


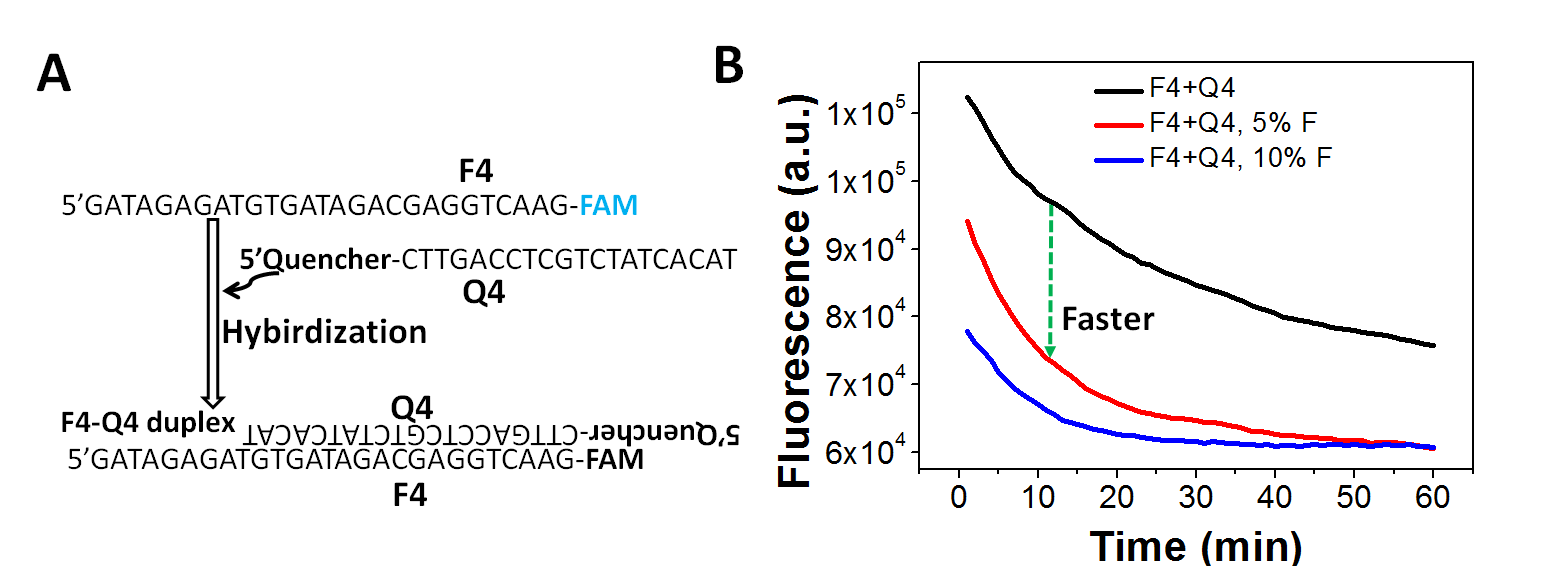


**Figure S5.** **Demonstration that formamide can significantly accelerate nucleic acid hybridization.** A) Scheme of hybridization of two linear complementary oligonucleotides, F4 and Q4. After hybridization, fluorescence of FAM labeled on 3’ end of F4 was quenched by the Quencher labeled on 5’ end of Q4. B) Real-time fluorescent kinetic monitoring right after Q4 was added into F4 solution, in presence of 0%, 5%, and 10% formamide, respectively. The experiments were carried out at 25 ^o^C, in 1×TNaK buffer. Q4 and F4 were designed without any self-folding structures under the experimental condition.

1. **Formamide softens nucleobase pairs.**

As shown in **Figure S6B**, melting temperature (Tm) of the reporter duplex (F1-Q1) decreases about 5 ^o^C when formamide increased every 10% volume concentration, suggesting formamide is weakening the duplex stability, more precisely, the hydrogen-bond binding energy between a nucleobase pair.


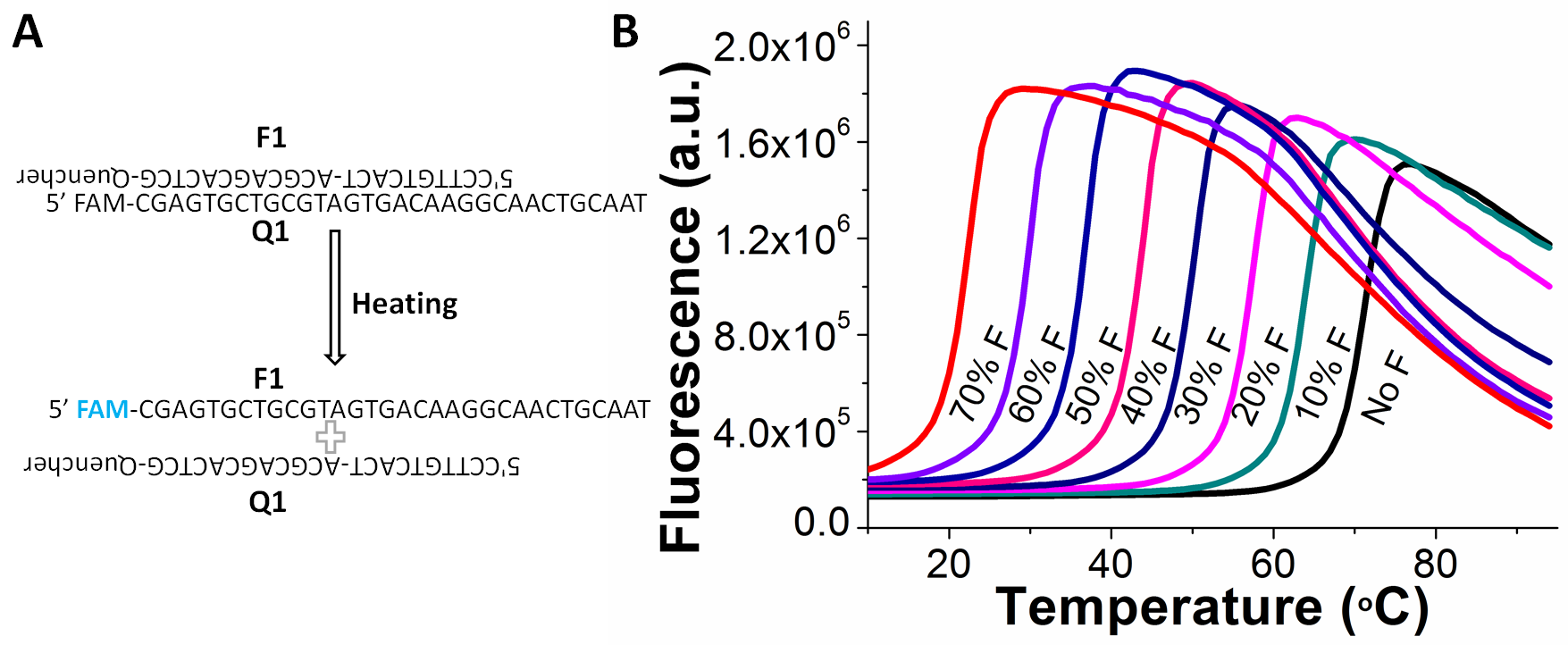


**Figure S6.** A) Scheme of melting temperature measurement of F1-Q1 duplex. B) Melting curves for the F1-Q1 duplex obtained in 1×Iso buffer in presence of various volume concentrations of formamide.

1. **Formamide revives toehold-mediated strand exchange reactions inhibited by small scale self-folding structures at desired functional temperatures.**

As shown in **Figure S7A** and **S7B** (red curve), a linear sequence, T_linear_ (5’-AAGGTAGCGGTTGACATAGTGGACAGG-3’), can initial a fast toehold mediated strand exchange reaction that displaces the Q5 sequence away from F5 sequence. However, when the toehold domain of T_linear_ (5’-AAGGTAGC-3’) contains self-folding structures, forming T_structured1_ or T_structured2_, the strand exchange rates (**Figure S7B**, blue and green curves) become much slower than that of T_linear_. The reaction in presence of T_structured2_ is even too slow to observe. It indicates that self-folding structures can indeed inhibit the toehold binding and then the whole strand exchange reaction. And the more stable the structure is, the more inhibition it will bring to the reaction. While when 25% formamide is imported into the same series of reactions, great acceleration in strand exchange rate is observed in presence of either T_structured1_ or T_structured2_ (**Figure S7C**, blue and green curves), much closer to the one in presence of T_linear_. It means formamide is weakening self-folding and narrowing the differences between linear (T_linear_) and self-folding structures (T_structured1_ and T_structured2_). Especially, the curve gotten from T_structured1_ even overlaps with the one gotten from T_linear_, indicating 25% formamide is already strong enough to completely melt the three base-pair structure in T_structured1_. ***Notably***, althrough formamide can generally more or less accelerate the strand exchange reactions triggered by T_structured1_ and T_structured2_, its acceleration function is not obviously observed in presence of T_linear_. It is understandable. Here the toehold domain of T_linear_ is designed to contain eight bases, which is already long enough to provide an ideal maximum strand exchange rate without formamide. In presence of formamide, it on one hand increases hybridization rate by enrichment, but on the other hand weakens toehold binding stability by softening base pairs. Therefore, for a strand exchange reaction in presence of an ideally designed trigger (e.g. T_linear_), a balanced function brought by the conflict enrichment and softening may not be definitely positive. It should be highly dependent on the toehold binding energy which is determined by its GC% and lengths.


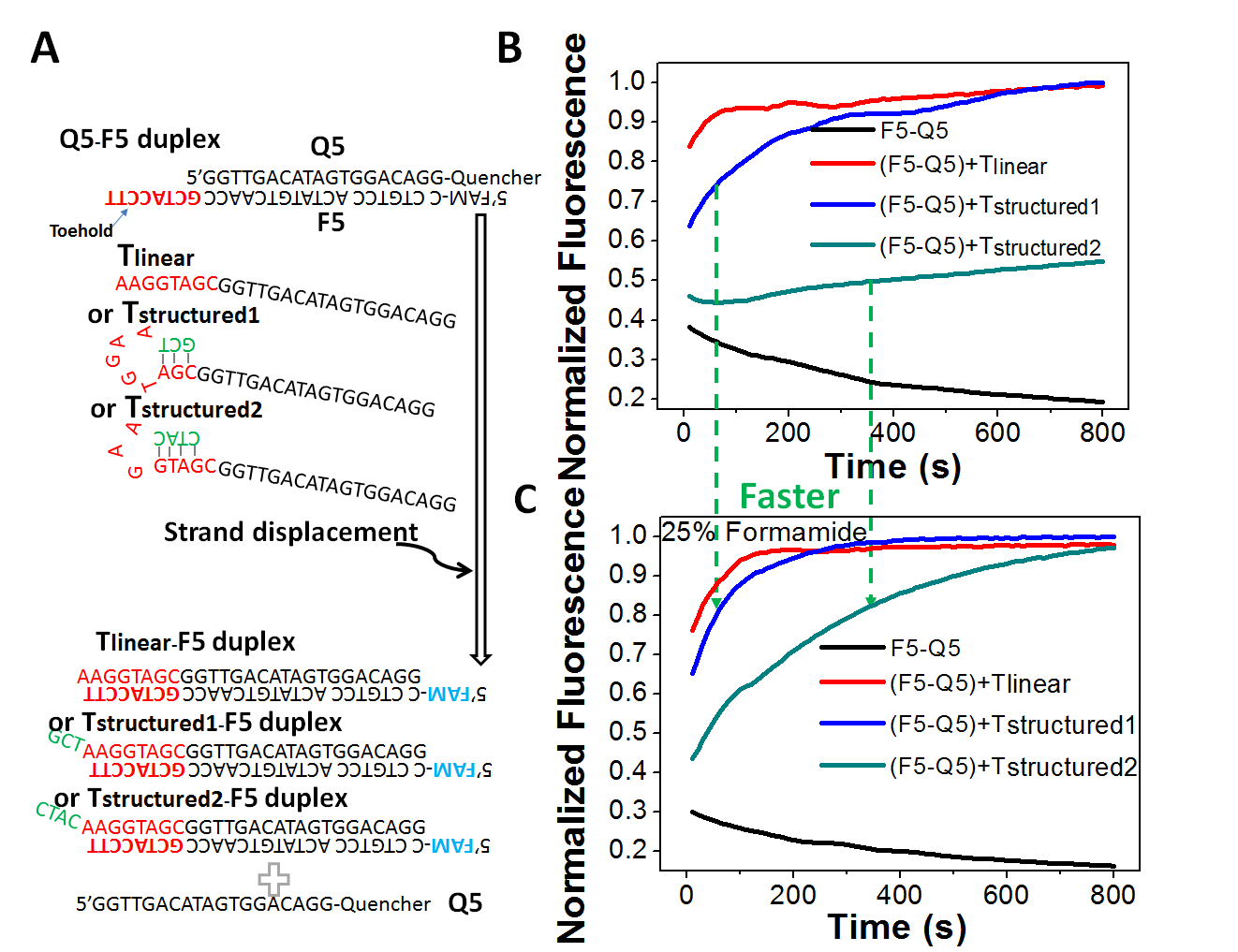


**Figure S7.** **Demonstration that formamide can significantly accelerate toehold-mediated strand exchange reaction inhibited by small scale self-folding structures.** A) Scheme of toehold mediated strand exchange reaction triggered by T_linear_, T_structured1_, and T_structured2_, respectively. The three triggers hold the same linear sequence of 5’-AAGGTAGC GGTTGACATAGTGGACAGG-3’ that can displace Q5 away from F5. The segment in red is toehold domain and the segment in black is branch migration domain. After strand exchange, fluorescence of FAM labeled on 5’ end of F5 is quenched by the Quencher labeled on 3’ end of Q5. B) Real-time fluorescent kinetic monitoring right after T_linear_, T_structured1_, or T_structured2_ wasadded into F5-Q5 duplex solution. C) Real-time fluorescent kinetic monitoring right after T_linear_, T_structured1_, or T_structured2_ was added into F5-Q5 duplex solution, in presence of 25% formamide. The experiments were carried out at 25 ^o^C, 1×TNaK buffer. Q5, F5, and T_linear_ were designed without any self-folding structure under the experimental condition. T_structured1_ and T_structured2_ were designed with three and four self-folding base pairs in the toehold domain, respectively. This Figure was the same with **Figure 3** in main text.

1. **Formamide revives a regular CHA reaction inhibited by small scale self-folding structures at desired functional temperature.**

Briefly, the effect of formamide on a regular CHA at 37 ^o^C is very similar to that on a one step toehold mediated strand exchange reaction (**Figure S7**).

In detail, as shown in **Figure S8A-S8C**, a linear sequence, C_linear_ (5’-**CGGTTGCT**TCTCTATCATTATCTTCC-3’), can serve as an ideal linear catalyst that triggers a high signal-to-background regular CHA reaction at 37 ^o^C. However, when the toehold domain of C_linear_ (5’- **CGGTTGCT** -3’) contains self-folding structures, forming C_structured1_ or C_structured2_, the initial CHA reaction rates (**Figure S8C**, green and blue curves) become much slower than that of C_linear_. The reaction in presence of C_structured1_ is even too slow to observe. Very similar to the condition in one step strand exchange reaction (**Figure S7**), self-folding structures can inhibit the toehold binding and then the whole CHA reaction. And the more stable the structure is, the more inhibition it will bring to the reaction. While when 20% formamide is imported into the same series of reactions, great acceleration in CHA rate is observed in presence of either C_structured1_ or C_structured2_ (**Figure S8D**, green and blue curves), much closer to the one in presence of C_linear_. It means formamide is weakening self-folding and narrowing the differences between linear (C_linear_) and self-folding structures (C_structured1_ and C_structured2_). Especially, the curve gotten from C_structured2_ even overlapps with the one gotten from C_linear_, indicating 20% formamide is already strong enough to completely melt the three base-pair structure in C_structured2_.

***Again***, althrough formamide has generally more or less accelerated the strand exchange reactions triggered by C_structured1_ and C_structured2_, its acceleration function is not obviously observed in presence of C_linear_. The explanation should be as same as that for one step strand exchange reaction. Therefore for a CHA reaction in presence of an ideally designed catalyst (e.g. C_linear_), a balanced function brought by the conflict enrichment and softening may not be definitely positive. It should be highly dependent on the toehold binding energy which is determined by its GC% and lengths.


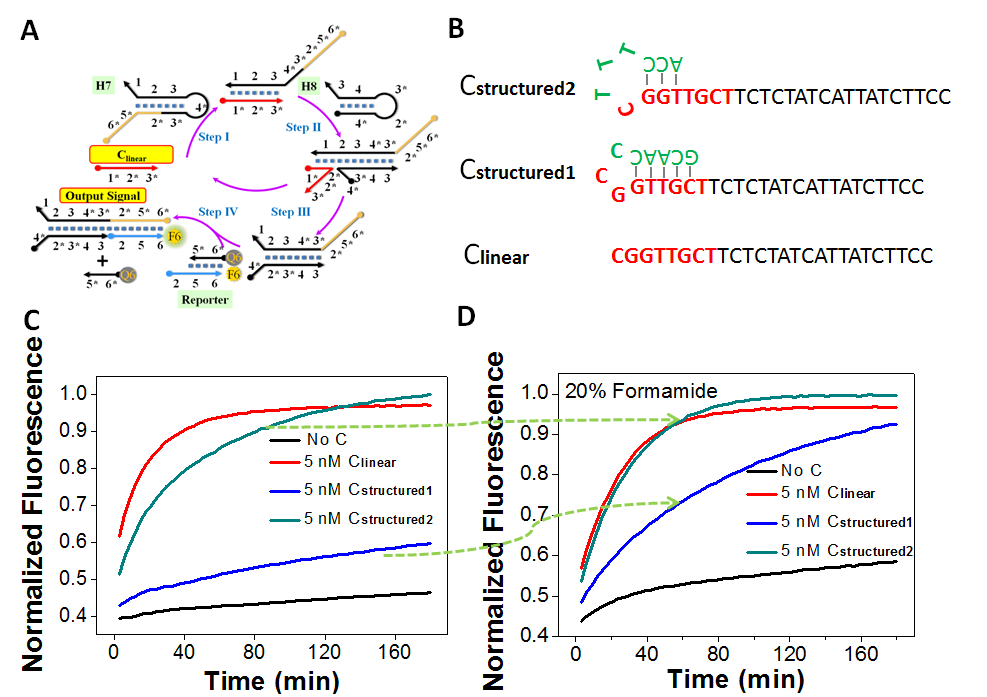


**Figure S8.** **Demonstration that formamide can significantly accelerate CHA reaction inhibited by small scale self-folding structures.** A) Scheme of a regular CHA (running at 37 ^o^C) composed of C_linear_, H7, H8, and F6-Q6 duplex. The pathway of a regular CHA is same to that of HT-CHA (**Figure 1A**), but the sequences of all components are shorter than those of HT-CHA. The detailed designing principles for both regular CHA and HT-CHA are listed in our previous publications^S1^. B) Sequences and structures of three catalysts, C_linear_, C_structured1_, and C_structured2_, respectively. The three catalysts hold the same linear sequence of 5’-**CGGTTGCT**TCTCTATCATTATCTTCC-3’ that can trigger the CHA reaction at 37 ^o^C. The segment in red is toehold domain and the segment in black is branch migration domain of the catalyst. After CHA reaction, fluorescence of FAM labeled on 5’ end of F6 is quenched by the Quencher labeled on 3’ end of Q6. C) Real-time fluorescent kinetic monitoring right after C_linear_, C_structured1_, or C_structured2_ was added into H7, H8, F6-Q6 duplex mixture. This figure is exactly the same as **Figure 4B and S1B**. D) Real-time fluorescent kinetic monitoring right after C_linear_, C_structured1_, or C_structured2_ was added into H7, H8, F6-Q6 duplex mixture, in presence of 20% formamide. This Figure is exactly the same with **Figure 4C**. The experiments were carried out at 37 ^o^C, in 1×TNaK buffer. C_linear_ was designed without any self-folding structure under the experimental condition. C_structured1_ and C_structured2_ were designed with three and five self-folding base pairs in the toehold domain, respectively.

1. **Formamide widens efficient region of a regular CHA to cooler circumstances than desired functional temperature.**

Being very similar to what happens in a HT-CHA system, a regular CHA (e.g. composed of C_linear_, H7, H8, and F6-Q6 duplex used in **Figure S8A**) will also gradually lose its catalytic efficiency when the running temperature shifts lower, especially much lower than the desired 37 ^o^C (**Figure S9. e.g. 10 ^o^C**). The reaction could be also explained by two inhibition mechanisms. First, which will definitely happen to any CHA set, automatic dissociation of C_linear_ from H7-H8-C_linear_ complex (Step III of a CHA reaction) will be slowed down due to the binding affinity between 1 and 1* domains (eight bases) becomes stronger at lower temperature. Second, which happens to this CHA set (C_linear_, H7, H8, and F6-Q6) but may not definitely to others, some unexpected self-folding structures start appearing at lower temperature in some linear-to-be intermediates in CHA pathway (e.g 4*-3*-2*-5*-6* segment of H7). According to the above discussion (**Figure S5-S8**), both inhibition mechanisms belong to unexpected folding structures, which might be more or less relieved by importing formamide in the reaction. Therefore, a significant recovery in catalytic reaction rate of the CHA at 10 ^o^C is observed in presence of 30 % formamide (**Figure S9**).


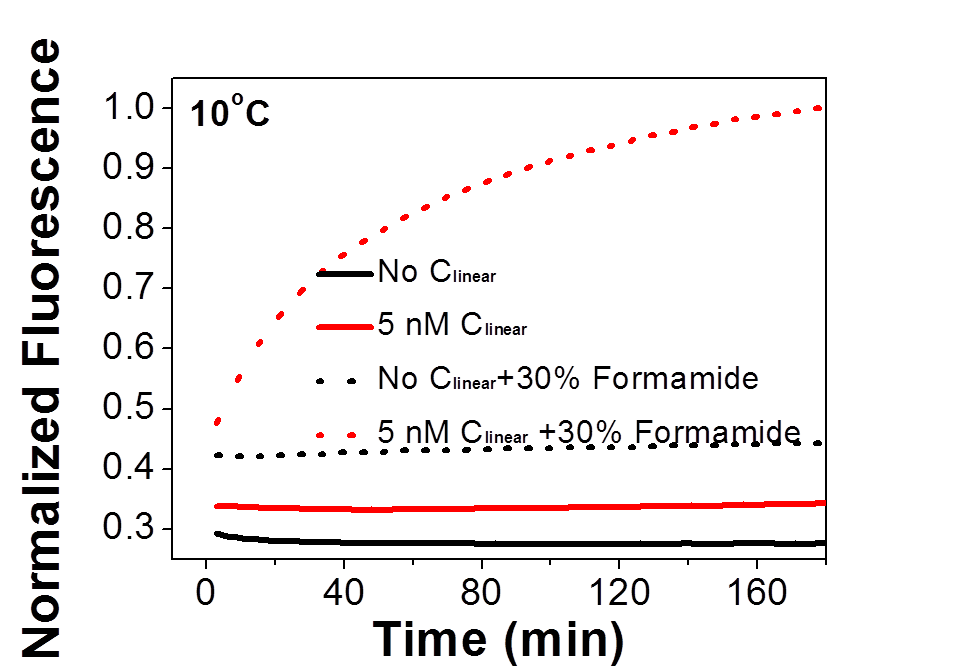


**Figure S9**. Fluorescent responses of the regular CHA used in **Figure S8** without and with 30% formamide at 10 °C with and without 5 nM C_linear_. The experiments were carried out in 1×TNaK buffer.

**
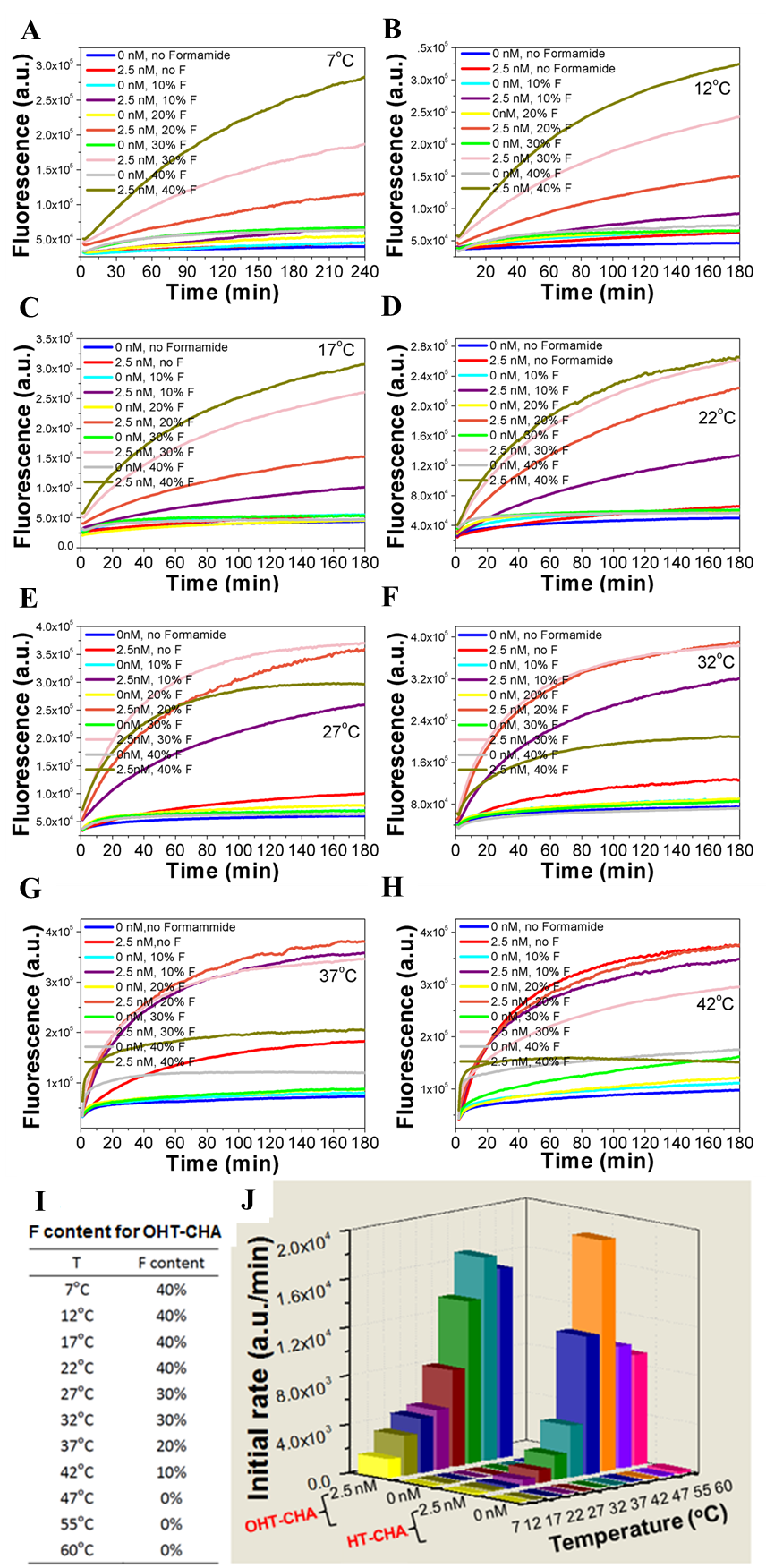
**

**Figure S10.** (A-H)Formamide concentration (volume) dependence in OHT-CHA1 (with and without C1) carried out from 7 ^o^C to 60 ^o^C, with 5 ^o^C as an interval. (I) Optimized formamide content for OHT-CHA1 at different temperatures. (J) 3D bar graph of initial rates (ΔRFU/Δt0-30min) of OHT-CHA1 and HT-CHA1 at each different temperature under respective best formamide content listed in Figure S10 I with and without C1. Calculation is based on fluorescent curves in Figure S10 A-H.

**
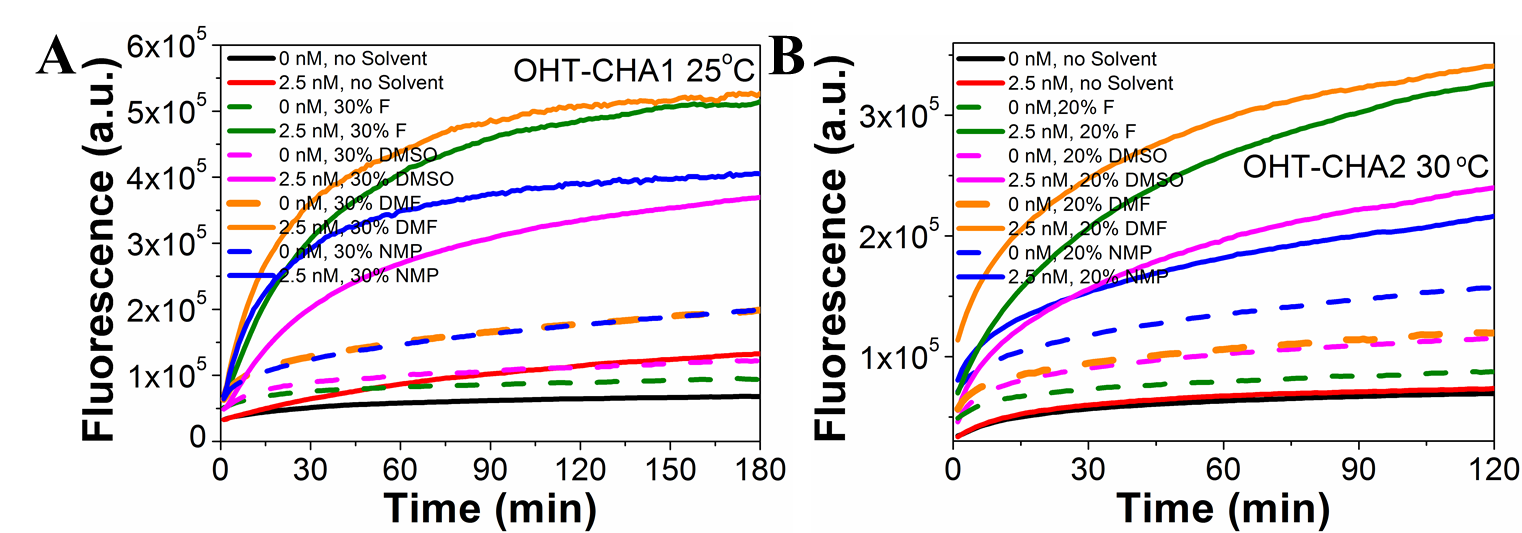
**

**Figure S11.** (A) Fluorescent responses of OHT-CHA1 at 25 °C with and without catalyst, in presence of 30% different amide solvents. (B) Fluorescent responses of OHT-CHA2 at 30 °C with and without catalyst, in presence of 20 % different amide solvents.


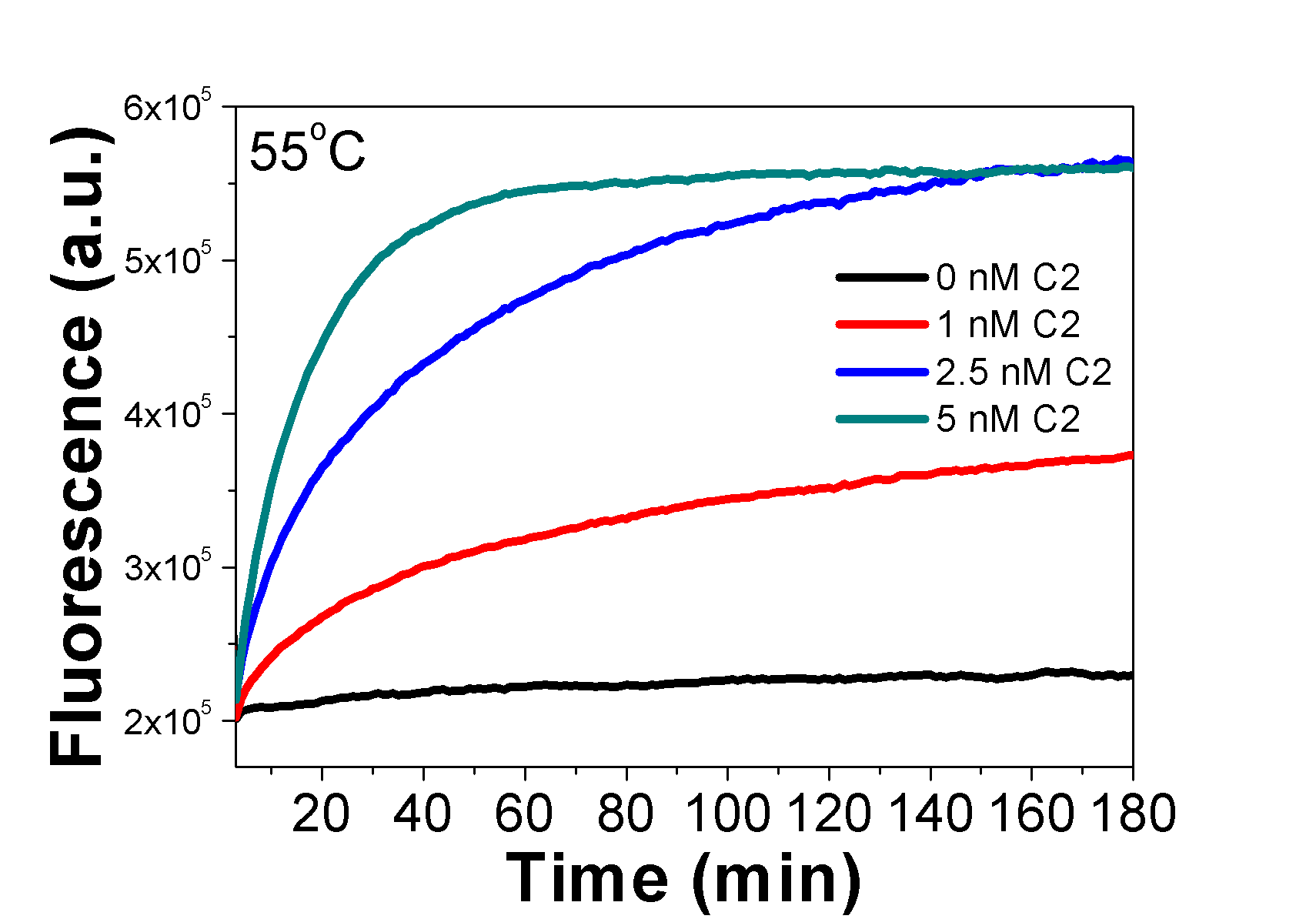


**Figure S12.** Fluorescent responses of another HT-CHA2 at 55 °C, with and without catalyst C2. The HT-CHA2 components used were C2, H3, H4, F2, Q2, which were the same as those used in **Figure 5A**.


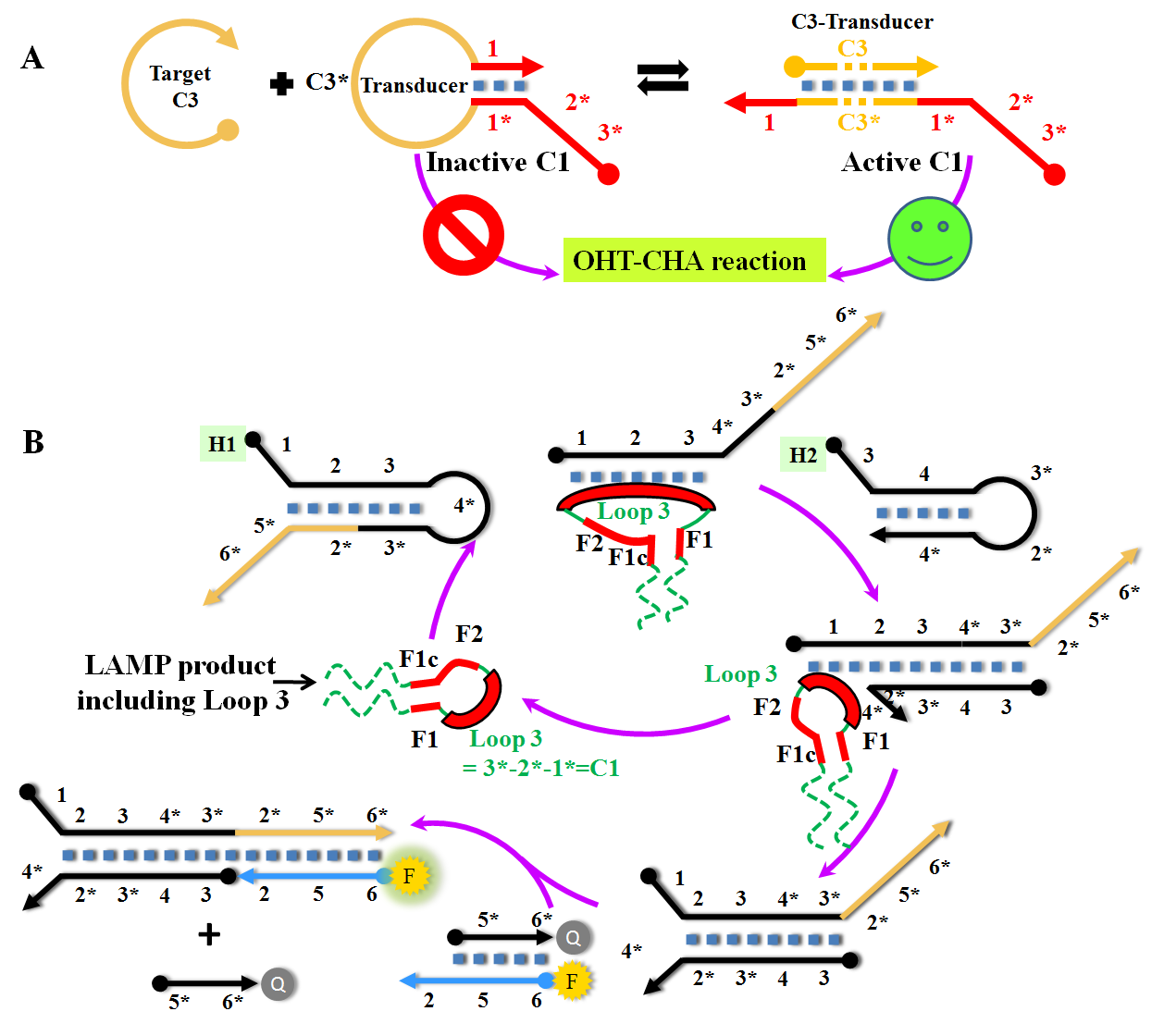


**Figure S13. (A)** Scheme of transducer triggered OHT-CHA1 circuits with target C3 as input. In the transducer hairpin, toehold domain of C1 (1*) was trapped in the stem of transducer. C1 was thus no longer available to trigger an active OHT-CHA1 reaction. In presence of C3, hybridization between C3 and C3* (loop sequence of transducer) could break up the transducer stem, releasing 1* domain free to trigger an active OHT-CHA1 reaction. This method allowed OHT-CHA being able to detect those sequences non-relevant to its components. The results proving this scheme were shown in **Figure 5B**. **(B)** Scheme of OHT-CHA1 triggered directly by Loop 3 in LAMP products.


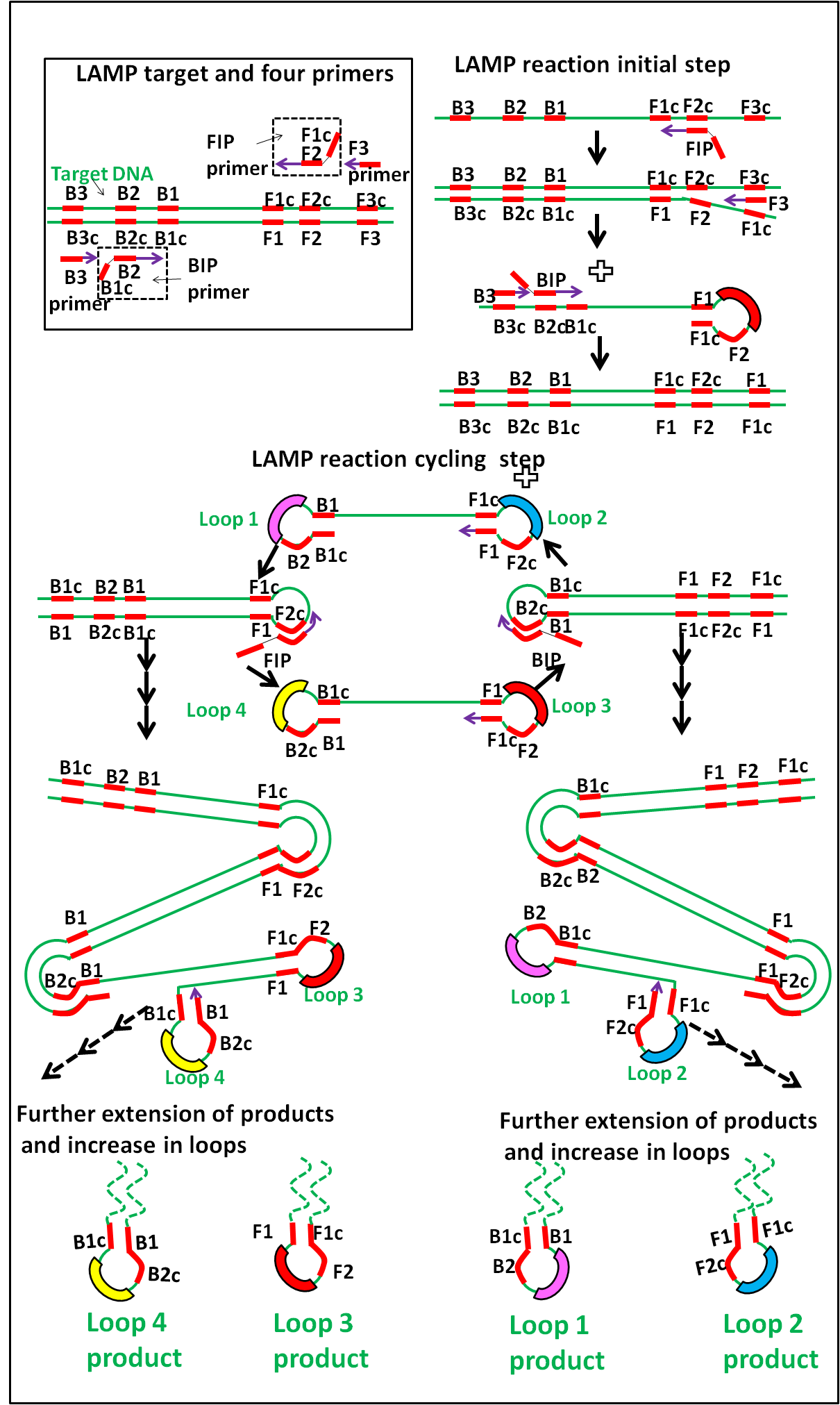


**Figure S14.** Scheme for LAMP reaction drawn according to the pathway suggested by original paper. ^S3^


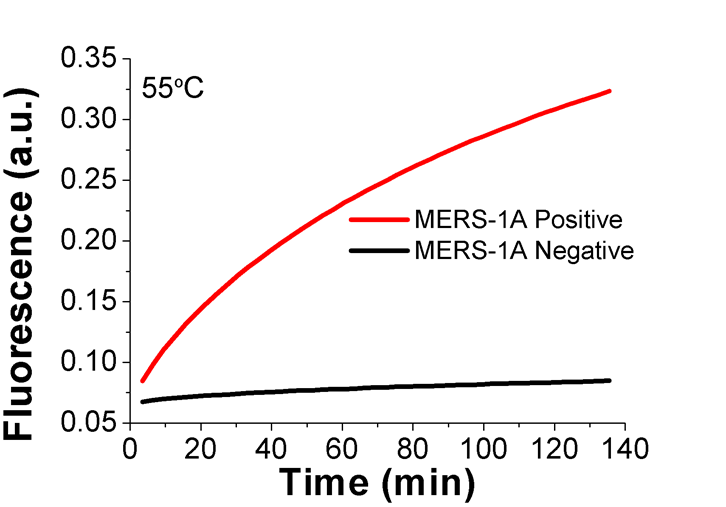


**Figure S15.** Using OHT-CHA1 as an end-point detector for LAMP products. Fluorescent responses of OHT-CHA to LAMP products amplified from synthetic MERS-1A DNA negative (0 copy) and positive (2000 copies, 6×10^-17^ M) samples. Data points were collected on a professional real-time PCR system at 55 °C.

**
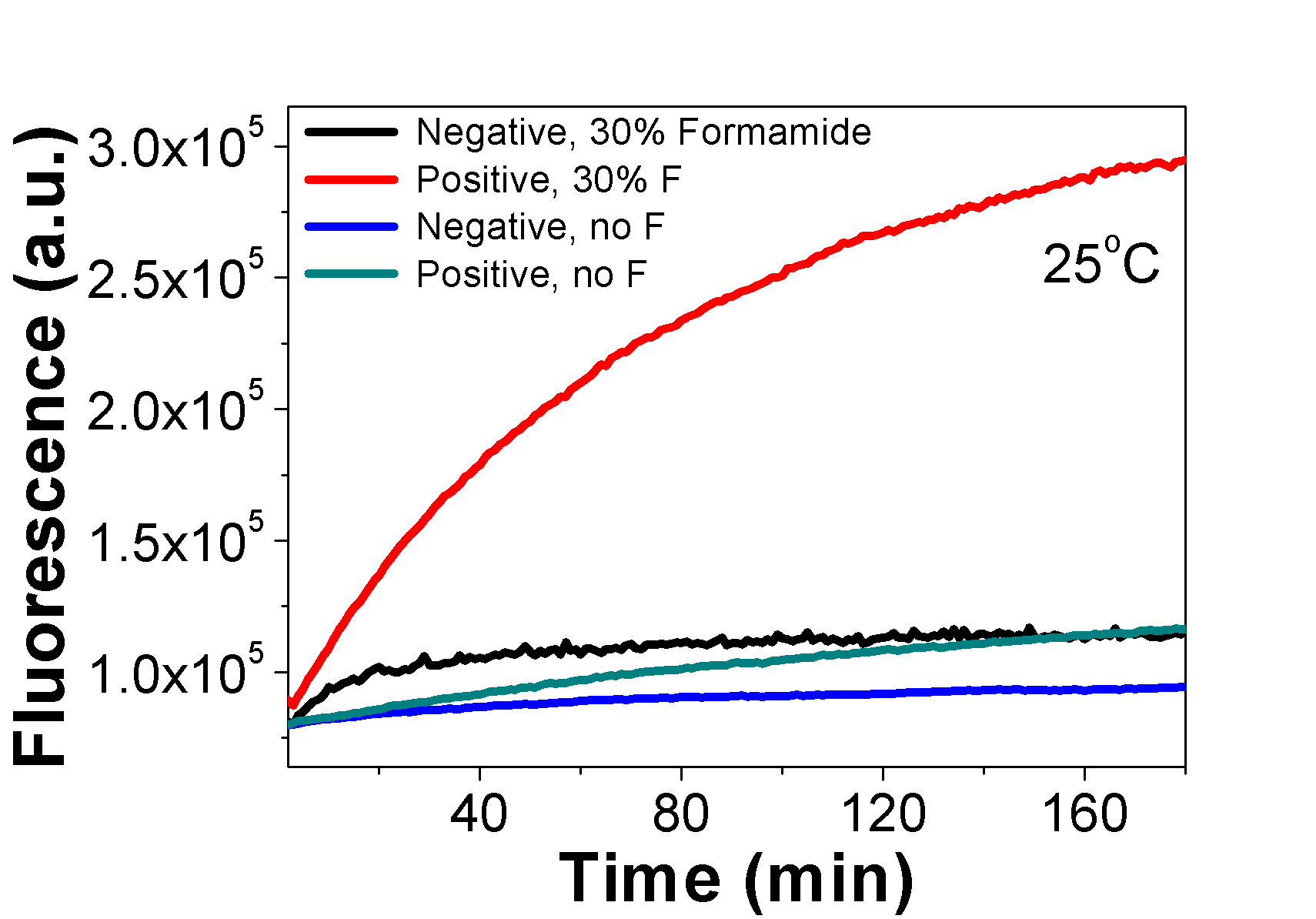
**

**Figure S16.** Fluorescent responses of HT-CHA1 (without formamide) and OHT-CHA1 (with 30% formamide) to LAMP products amplified from MERS-1A DNA negative (0 copy) and positive (2000 copies, 6×10^-17^ M) samples. The fluorescent reading was performed at 25 ^o^C.


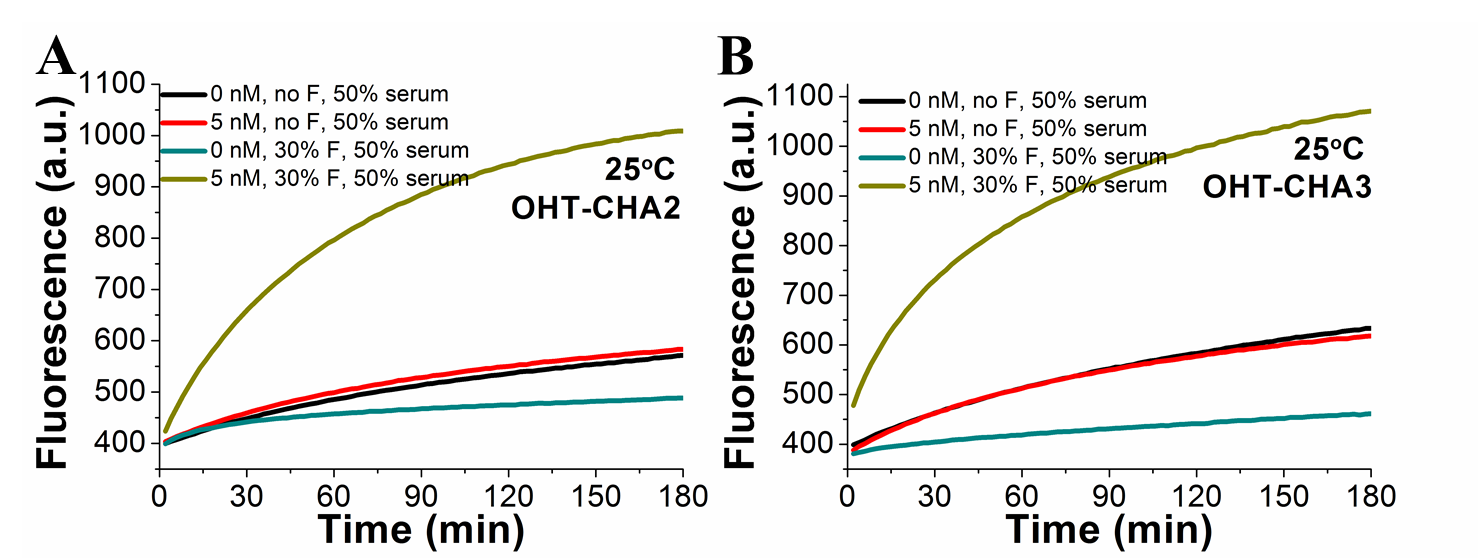


**Figure S17**. Fluorescent responses of OHT-CHA2 (A) and OHT-CHA3 (B) in presence of 50% fetal bovine serum, without and with 30% formamide at 25 °C, with and without 5 nM catalyst..

| **Name** | **Sequence 5’-3’** | **Label** | **Note** |
| --- | --- | --- | --- |
| **H1** | AGCCAATTT-GCAACTGCAAT-CAGCGCTGA-CCATCCTGCTAGCA-TCAGCGCTG-ATTGCAGTTGC-CTTGTCACT-ACGCAGCAC  1-2-3-4*-3*-2*-5*-6* |  | Sequences used in Sequences used in OHT-CHA1 (Figure 2, 5B, 6, 7, S2-S4, S6 S10-S11A, and S15-S16.  The CHA was designed for Loop3 (C1) of MERS-1A LAMP amplicons  3’ end of H1, H2, F1 used in Figure 6 A was invert-dT modified |
| **H2** | CAGCGCTGA-TGCTAGCAGGATGG-TCAGCGCTG-ATTGCAGTTGC-CATCCTGCTAGCA  3-4-3*-2*-4* |  |
| **F1** | CGA-GTGCTGCGT-AGTGACAAG-GCAACTGCAAT  CGA-6-5-2 | 5’FAM |
| **Q1** | C-CTTGTCACT-ACGCAGCAC-TCG  C-5*-6*-TCG | 3’BHQ1 |
| **C1** | GC-TCAGCGCTG-ATTGCAGTTG CAAATTGGCT  GC-3*-2*-1* |  |
| **C1_Mis1_** | GCTCAGCGCTGATTGCAGTTG CAAAT**A**GGCT  GC-3*-2*-1mis1* |  |
| **C1_Mis2_** | GCTCAGCGCTGATTGCAGTT**C** CAAATTGGCT  GC-3*-2*-1mis2* |  |
| **C3** | TCGCTTATCGTTTAAGCAGCTCTGCGCTACTATGGGTCC  Underline of **C3** was complementary to underline of **Transducer** |  |
| **Transducer** | CTCAGCGCTGATTGCAGTTGCAAATTGGCTGCGGACCCATAGTAGCGCAGAGCTGCTTAAACGATAAGCGAGCCAATTTGCAAC |  |
| **H3** | CGTGGAGGC-GATCACACCG-CAGACGTTGA-CCACGCTGCTAGCA-TCAACGTCTG-CGGTGTGATC-CCTTGTCATA-CGCAGCAC  1-2-3-4*-3*-2*-5*-6* |  | Sequences used in OHT-CHA2 (Figure 5A), HT-CHA2 (Figure S12), and OHT-CHA2 (Figure S11B and S17A) |
| **H4** | CAGACGTTGA-TGCTAGCAGCGTGG-TCAACGTCTG-CGGTGTGATC-CCACGCTGCTAGCA  3-4-3*-2*-4* |  |
| **F2** | CGA-GTGCTGCG-TATGACAAGG-GATCACACCG  CGA-6-5-2 | 5’FAM |
| **Q2** | C-CCTTGTCATA-CGCAGCAC-TCG  C-5*-6*-TCG | 3’BHQ1 |
| **C2** | TCAACGTCTG-CGGTGTGATC-GCCTCCACG  3*-2*-1* |  |
| **H5** | GAGCACGAT-CATCATCCAGAG-AGAATTATCGAG- CCATCCTCCTACCC-CTCGATAATTCT-CTCTGGATGATG- CCTTGTCAC-TACGCAGCAC  1-2-3-4*-3*-2*-5*-6* |  | Sequences used in OHT-CHA3  (Figure 8 and Figure S17B)  The CHA was designed for Loop3 (C4) of ZEBOV LAMP amplicons |
| **H6** | AGAATTATCGAG-GGGTAGGAGGATGG-CTCGATAATTCT-CTCTGGATGATG-CCATCCTCCTACCC  3-4-3*-2*-4* |  |
| **F3** | CGA GTGCTGCGTA-GTGACAAGG-CATCATCCAGAG  CGA-6-5-2 | 5’FAM |
| **Q3** | CCTTGTCAC-TACGCAGCAC-TCG  5*-6*-TCG | 3’BHQ1 |
| **C4** | CAC-CTCGATAATTCT-CTCTGGATGATG-ATCGTGCTC  CAC-3*-2*-1* |  |
| **F4** | ATGTGATAGACGAGGTCAAG | 3’FAM | Sequences used in  Figure S5 |
| **Q4** | CTTGACCTCGTCTATCACAT | 5’BHQ1 |
| **T_linear_** | AAGGTAGC-GGTTGACATAGTGGACAGG |  | Sequences used in  Figure 3,S7 |
| **T_stuctured1_** | GCT-AAGGTAGC-GGTTGACATAGTGGACAGG |  |
| **T_stuctured2_** | CTAC-AAGGTAGC-GGTTGACATAGTGGACAGG |  |
| **F5** | CCTGTCCACTATGTCAACC-GCTACCTT | 5’FAM |
| **Q5** | GGTTGACATAGTGGACAGG | 3’BHQ1 |
| **C_linear_** | CGGTTGCT-TCTCTATC-ATTATCTT  1*-2*-3* |  | Sequences used in  Figure 4, S1,S8, and S9 |
| **C_structured1_** | GCAACC-CGGTTGCT-TCTCTATC-ATTATCTT  GCAACC-1*-2*-3* |  |
| **C_structured2_** | ACCTTT-CGGTTGCT-TCTCTATC-ATTATCTT  ACCTTT -1*-2*-3* |  |
| **H7** | GACCTCGT-CTATCACA-TCTCTATC-ATTATCTT-CCTAGTGTTAAC-AAGATAAT-GATAGAGA-AGCAACCG-AC  6*-5*-2*-3*-4*-3-2-1 -AC |  |
| **H8** | CCTAGTGTTAAC-TCTCTATC-ATTATCTT-GTTAACACTAGG-AAGATAAT  4*-2*-3*-4-3 |  |
| **F6** | GATAGAGA-TGTGATAG-ACGAGGTC-AAG  2-5-6-AAG | 3’FAM |
| **Q6** | CTT-GACCTCGT-CTATCACA-T  CTT-6*-5* | 5’BHQ1 |
| **LAMP primer sequences** | | | |
| **MERS-1A-F3** | TCAACGTCTGCGGTGTGATCGCCTGCACG | | MERS-1A LAMP primers used in Figure 6,S15, S16, |
| **MERS-1A-B3** | CGCAAAGTTAGAAAGTGATGG | |
| **MERS-1A-FIP** | AAGCATTAGTGGGGGCAAGCCCCACTACTCCCATTTCG | |
| **MERS-1A-BIP** | ATGCGCACTACACATACTGATATTTGTACAATCTCTTCACTACAATGA | |
| **MERS-1A-LP** | GGTGTCTACATTAGTATGTCACTTGTATTAG | |
| **RPOB-F3** | GCATGTCGCGGATGGAG | | RPOB LAMP primers used in Figure 7 |
| **RPOB-B3** | CGCTCACGTGACAGACCG | |
| **RPOB-FIP** | CTTGATCGCGGCGACCACCGAGCGGATGACCACCCA | |
| **RPOB-BIP** | CAGCCAGCTGAGCCAATTCATGGACCAGACAGTCGGCGCTTGTG | |
| **EBOLA-F3** | AGACAGCATTCAAGGGATG | | ZEBOV  LAMP primers used in Figure 8 |
| **EBOLA-B3** | CCTTTTTTCAAGGTCGGACA | |
| **EBOLA-FIP** | CTCCTTGATTGACGGTACTCACCGACACGACCACCATGTTC | |
| **EBOLA-BIP** | CTCACAAGTGCGCGTTCCTAATGTCTTTAGGTGCTGGAG | |
|  | | | |

**Table S1**: Oligonucleotides used in this paper. All labeled sequences were purified with high-pressure liquid chromatography. All unlabeled sequences were polyacrylamide gel electrophoresis purified.

| **Figure** | **CHA or nucleic acid components** | **Operating**  **temperature** | **Amide** | **Input** | **Instrument** |
| --- | --- | --- | --- | --- | --- |
| **Figure 2B** | [H1]=1/4[H2]=  [F1]=1/2[Q1]=  50 nM | 55 ^o^C | No  formamide | 0 nM or 2.5 nMC1 | Real-time PCR  system |
| **Figure 2C** | [H1]=1/4[H2]=  [F1]=1/2[Q1]=  50 nM | 20 ^o^C | No or 40%  formamide | 0 nM or 2.5 nMC1 | Real-time PCR  system |
| **Figure 3** | [F5]=100 nM  [Q5]=120 nM | 25 ^o^C | No or 25%  formamide | 0nM, 120 nM T_linear_, 120nM T_stuctured1_, or 120 nM T_structured2_ | Real-time PCR  system |
| **Figure 4** | [H7]=1/4[H8]=  [F6]=1/2[Q6]=  50 nM | 37^o^C | No or 20%  formamide | 0 nM,5 nM C_linear_, 5nM C_structured1_ or  5 nM C_structured2_ | Real-time PCR  system |
| **Figure 5A** | [H3]=1/4[H4]=  [F2]=1/2[Q2]=  50 nM | 17 ^o^C | No or 30 %  formamide | 0 nM or 2.5 nMC1 | Real-time PCR  system |
| **Figure 5B** | [H1]=1/4[H2]=  [F1]=1/2[Q1]=  50 nM  [Transducer]=3 nM | 25 ^o^C | 30 %  formamide | 0 nM or  3 nM C3 | Real-time PCR  system |
| **Figure 6A** | [H1]= [H2]=  [F1]= [Q1]=  200 nM | 55 ^o^C | No  formamide | LAMP amplicons from 0 or 2000 copy MERS-1A | Real-time PCR  System  (real-time) |
| **Figure 6B** | [H1]=1/4[H2]=  [F1]=1/2[Q1]=  50 nM | 19 °C-21 °C | 30 %  formamide | LAMP amplicons from 0 or 2000 copy MERS-1A | fluorescence (FL) spectrum  (end-point) |
| **Figure 6C** | [H1]= [H2]=  [F1]= [Q1]=  200 nM | 6.5 °C-12 °C | 30 %  formamide | LAMP amplicons from 0 or 2000 copy MERS-1A | Portable PL detector  (end-point) |
| **Figure 7** | [H1]=1/4[H2]=  [F1]=1/2[Q1]=  50 nM | 25 °C | 30 %  formamide | LAMP amplicons from various MERS-1A | Real-time PCR  system |
| **Figure 8** | [H5]=1/4[H6=  [F3]=1/2[Q3]=  50 nM | 25 °C | 25 %  formamide | LAMP amplicons from 0 or 2000 copy ZEBOV | Portable real-time PCR system |
| **Figure S1** | [H7]=1/4[H8]=  [F6]=1/2[Q6]=  50 nM | 37^o^C | No  formamide | 0 nM,5 nM C_linear_, 5nM C_structured1_ or  5 nM C_structured2_ | Real-time PCR  system |
| **Figure S2** | [H1]=1/4[H2]=  [F1]=1/2[Q1]=  50 nM | 47 ^o^C, 55 ^o^C, and 60 ^o^C | No  formamide | 0 nM or 2.5 nMC1 | Real-time PCR  system |
| **Figure S4** | [H1]=1/4[H2]=  [F1]=1/2[Q1]=  50 nM | 25 °C | 30 %  formamide | Various C1 and C1_mis1_ | Real-time PCR  system |
| **Figure S5** | [F4]= 50 nM | 25 ^o^C | No, 5%, or 10% formamide | [Q4]=100 nM | Real-time PCR  system |
| **Figure S6** | [F1]= [Q1]=50 nM | 25 ^o^C | Various  formamide | Melting curve | Real-time PCR  system |
| **Figure S7** | [F5]=100 nM  [Q5]=120 nM | 25 ^o^C | No or 25%  formamide | 0nM, 120 nM T_linear_, 120nM T_stuctured1_, or 120 nM T_structured2_ | Real-time PCR  system |
| **Figure S8** | [H7]=1/4[H8]=  [F6]=1/2[Q6]=  50 nM | 37^o^C | No or 20%  formamide | 0 nM,5 nM C_linear_, 5nM C_structured1_ or  5 nM C_structured2_ | Real-time PCR  system |
| **Figure S9** | [H7]=1/4[H8]=  [F6]=1/2[Q6]=  50 nM | 10^o^C | 30%  formamide | 0 nM,5 nM C_linear_, 5nM C_structured1_ or  5 nM C_structured2_ | Real-time PCR  system |
| **Figure S10** | [H1]=1/4[H2]=  [F1]=1/2[Q1]=  50 nM | Various | Various  formamide | 0 nM or 2.5 nMC1 | Real-time PCR  system |
| **Figure S11** | [H1]=1/4[H2]=  [F1]=1/2[Q1]=  50 nM | 25 °C | No, or formamide, DMSO, DMF, NMP | 0 nM or 2.5 nMC1 | Real-time PCR  system |
| **Figure S12** | [H3]=1/4[H4]=  [F2]=1/2[Q2]=  50 nM | 55 °C | No  formamide | 0 nM or 2.5 nM C2 | Real-time PCR  system |
| **Figure S15** | [H1]= [H2]=  [F1]= [Q1]=  200 nM | 55 ^o^C | No  formamide | LAMP amplicons from 0 or 2000 copy MERS-1A | Real-time PCR  System  (end-point) |
| **Figure S16** | [H1]=1/4[H2]=  [F1]=1/2[Q1]=  50 nM | 25 °C | No or 30 %  formamide | LAMP amplicons from 0 or 2000 copy MERS-1A | Real-time PCR  system |
| **Figure S17** | [H1]=1/4[H2]=  [F1]=1/2[Q1]=  50 nM | 25 °C | No or 30 %  Formamide  50% fetal bovine serum | 0 nM or 5 nM C2(or C4) | Portable real-time PCR system |

**Table S2**: Reaction conditions used in experiments for each Figure.

**References in supporting information**

S1. Jiang, Y. S., Li, B., Milligan, J. N., Bhadra, S. & Ellington, A. D. Real-time detection of isothermal amplification reactions with thermostable catalytic hairpin assembly. *J. Am. Chem. Soc.* **135,** 7430-7433(2013).

S2. Bonner, J., Kung, G. & Bekhor, I. A method for the hybridization of nucleic acid molecules at low temperature. *Biochemistry*. **6,** 3650-3653(1967).

S3. Notomi, T. et al. Loop-mediated isothermal amplification of DNA. *Nucleic Acids Res*. **28,** e63(2000).
